# Formulation and Testing of Alginate Microbeads Containing *Salvia officinalis* Extract and Prebiotics

**DOI:** 10.3390/pharmaceutics17101308

**Published:** 2025-10-08

**Authors:** Krisztina Bodnár, Pálma Fehér, Zoltán Ujhelyi, Ádám Haimhoffer, Boglárka Papp, Dávid Sinka, Csongor Freytag, Eszter Fidrus, Krisztina Szarka, Gábor Kardos, Fruzsina Nacsa, Ildikó Bácskay, Liza Józsa

**Affiliations:** 1 Department of Pharmaceutical Technology, Faculty of Pharmacy, University of Debrecen, 4032 Debrecen, Hungary; bodnar.krisztina@pharm.unideb.hu (K.B.); feher.palma@pharm.unideb.hu (P.F.); haimhoffer.adam@pharm.unideb.hu (Á.H.); papp.boglarka@pharm.unideb.hu (B.P.); sinka.david@pharm.unideb.hu (D.S.); bacskay.ildiko@pharm.unideb.hu (I.B.); 2 Doctoral School of Pharmaceutical Sciences, University of Debrecen, 4032 Debrecen, Hungary; ujhelyi.zoltan@pharm.unideb.hu; 3 Department of Industrial Pharmaceutical Technology, Faculty of Pharmacy, University of Debrecen, Rex Ferenc Utca 1, 4002 Debrecen, Hungary; 4 Department of Bioinformatics, One Health Institute, Faculty of Health Sciences, University of Debrecen, 4032 Debrecen, Hungary; freytag.csongor@etk.unideb.hu (C.F.); fidrus.eszter@etk.unideb.hu (E.F.); 5 Department of Infection Control and Hospital Epidemiology, One Health Institute, Faculty of Health Sciences, University of Debrecen, 4032 Debrecen, Hungary; szkrisz@med.unideb.hu; 6 Department of Planetary Health, One Health Institute, Faculty of Health Sciences, University of Debrecen, 4032 Debrecen, Hungary; kg@med.unideb.hu; 7 Institute of Metagenomics, University of Debrecen, 4032 Debrecen, Hungary; 8 MEDITOP Pharmaceutical Ltd., 2097 Pilisborosjeno, Hungary; fruzsina.nacsa@meditop.hu

**Keywords:** sage extract, encapsulation, pectin, inulin, anti-inflammatory effect, antioxidant effect

## Abstract

**Background/Objectives**: This study aimed to develop an advanced oral delivery platform for *Salvia officinalis* (*S. officinalis*) extract by co-encapsulating it with inulin and pectin in alginate-based microbeads, formulated via ionic gelation. **Methods**: The microbeads were comprehensively characterized, including the assessment of morphology, particle size, encapsulation efficiency, swelling behavior, in vitro dissolution, and enzymatic stability, and Caco-2 cell-based assays for cytocompatibility, permeability, and transepithelial electrical resistance. Antioxidant capacity and anti-inflammatory effects were also evaluated. **Results**: The resulting microbeads (~275 µm) achieved > 90% encapsulation efficiency and exhibited pronounced swelling (~90%). The release of *S. officinalis* constituents displayed pH sensitivity, with sustained release in simulated intestinal fluid, alongside significant enhancement of enzymatic stability. Encapsulation led to markedly improved permeability of bioactive compounds across Caco-2 monolayers, attributable to reversible modulation of tight junctions. Encapsulated extract retained potent antioxidant activity and significantly reduced pro-inflammatory cytokines. The formulation, across various concentrations, further promoted the growth and viability of *Lactobacillus* strains. **Conclusions**: Collectively, these findings demonstrate that alginate–inulin–pectin microbeads provide a multifunctional system for stabilizing *S. officinalis* extract, enabling controlled release, enhanced intestinal absorption, and maintained bioefficacy. Importantly, the formulation also promoted *Lactobacillus* viability, indicating a prebiotic effect and offering considerable potential for improved oral therapeutic applications.

## 1. Introduction

*Salvia officinalis* (sage, *S. officinalis*) is a perennial, rounded shrub native to the Middle East and Mediterranean, widely recognized for its rich and complex chemical composition and traditional medicinal use [1,2]. The extract of *S. officinalis* contains a diverse mixture of biologically active secondary metabolites whose synergistic interactions contribute to its therapeutic effects [3,4,5,6]. Phenolic acids, primarily rosmarinic acid and caffeic acid, typically constitute about 3.5–12% of the dry weight of the plant. Phenolic diterpenes, mainly carnosic acid and carnosol, are present in concentrations of approximately 2–6% *w*/*w* in dry extracts. Flavonoid derivatives, including apigenin, luteolin, kaempferol, and quercetin, also make up notable fractions of the extract. Additionally, the essential oil fraction, representing 0.1–2.8% of the aerial parts, contains monoterpenes such as 1,8-cineole, camphor, and α-thujone, alongside various sesquiterpenes [7,8,9,10,11,12]. The therapeutic activities of sage—including antioxidant, anti-inflammatory, antimicrobial, neuroprotective, and metabolic effects—are attributed to the combined and often synergistic action of these compounds [4,12]. Recent pharmacological research has confirmed the antidiabetic, anticancer, antimicrobial, and anti-inflammatory effects of the sage, alongside beneficial influences on cognitive function, memory, and preservation of food products [13,14,15]. This pharmacological profile reinforces the importance of considering not only individual metabolites, but their concerted action in complex plant extracts.

Carnosol was selected as a chemical marker and focus of this study due to its prominent antioxidant and anti-inflammatory activities, which have been well documented in the literature [16,17]. Its physicochemical properties, including moderate lipophilicity and stability under physiological conditions, render it suitable for encapsulation approaches aimed at targeted delivery and controlled release [18]. The emphasis on carnosol in quantitative assays such as encapsulation efficiency, in vitro release, enzymatic stability, and permeability reflects its significant contribution to the overall bioactivity of *S. officinalis* extracts. Although other secondary metabolites like rosmarinic acid are also important, carnosol’s specific balance of bioactivity and stability guided its selection as a reference compound in this work [19]. The concentration of carnosol in the extract could be reliably quantified by spectrophotometric methods (typically at 280–285 nm) [20]. The carnosol content in the extract was 55 μg/mg, and the spectrophotometric determination enabled us to monitor the encapsulation and release processes quantitatively and reproducibly.

Studies have demonstrated that sage extracts have been shown to stimulate the growth of probiotic bacteria such as Lactobacillus (e.g., *Lacticaseibacillus rhamnosus*), while also inhibiting certain pathogenic microorganisms, thereby promoting a healthier microbial balance in the gut. This prebiotic-like effect is attributed to the rich phytochemical profile of the plant, including polyphenols and terpenoids, which can selectively enhance the viability and proliferation of beneficial gut bacteria [21,22]. Additionally, animal studies demonstrate that administration of *S. officinalis* can help correct dysbiotic disorders, supporting the restoration of a balanced intestinal microbiome during colitis or after exposure to gut pathogens, all without adversely impacting normal microbiota. Furthermore, sage can modulate gut motility, exhibiting antidiarrheal and antispasmodic effects, potentially via smooth muscle relaxation mechanisms [23].

Among the natural polysaccharides, alginates have become widely used in drug delivery over the past three decades. Alginate, derived from seaweed, is a copolymer made up of β-d-mannuronate and α-l-guluronate. Alginate-based nanoparticles are biocompatible, biodegradable, and capable of protecting drugs from the harsh conditions of the gastrointestinal tract. Additionally, they can offer targeted delivery, controlled release, and sustainability. The mild gelation properties of the alginate, activated by divalent cations like Ca^2+^, have made it valuable in fields such as wound healing, tissue engineering, and orthopedics due to its low toxicity, biocompatibility, and osteoconductivity [24,25,26].

Inulin, a soluble dietary fiber, has garnered considerable interest due to its broad health benefits, particularly its ability to modulate the gut microbiota and enhance immune function [27]. As a prebiotic, inulin selectively stimulates the growth and activity of beneficial gut bacteria such as *Bifidobacterium* and *Lactobacillus*, leading to increased production of short-chain fatty acids (SCFAs) including acetate, propionate, and butyrate. These SCFAs play critical roles in maintaining intestinal barrier integrity, regulating immune responses, and suppressing inflammation. Inulin supplementation has been shown to improve microbial diversity and reduce pro-inflammatory bacterial populations, thus contributing to a healthier gut environment [28,29].

Similarly, pectin is a multifunctional polysaccharide known for its beneficial effects on digestion and cholesterol reduction, partly through increased bile acid excretion [30]. Beyond its role as a soluble fiber, pectin also exhibits prebiotic properties by fostering the growth of SCFA-producing bacteria and modulating the gut microbiota composition. The significant anti-inflammatory and immunomodulatory effects of pectin further contribute to maintaining gut homeostasis and preventing inflammation-related disorders. Together, inulin and pectin act as potent prebiotics that support the growth of beneficial microbes, enhance gut health, and contribute to systemic immune modulation [31,32]. In our encapsulation system, both inulin and pectin were utilized to enhance the structural integrity and functional properties of the alginate-based beads. Inulin, a fructan polysaccharide, contributes to the formation of hydrogels through physical crosslinking mechanisms. Its high degree of hydration and gel-forming ability improve the mechanical strength and stability of the beads, facilitating the controlled release of encapsulated bioactives [33]. Pectin, a galacturonic acid-rich polysaccharide, forms gels in the presence of divalent cations like calcium. When combined with alginate, pectin enhances the gel network’s density and elasticity, leading to improved encapsulation efficiency and protection of sensitive compounds [34].

Prebiotic interventions have been increasingly recognized for their role in managing metabolic disorders such as diabetes, and for modulating the gut microbiome—an effect that may be potentiated by synergistic combinations with plant-extracted phytonutrients. Different dietary intervention studies have been used for long run, for instance, sage leaf extracted and supplemented 3 months to diabetes and hyper-cholesterol individuals revealed lower levels of fasting glucose, total cholesterol and triglyceride [35,36]. Other studies showed that sage consumption led to improvements in memory in Alzheimer’s patients and healthy young and old persons, as it enhanced the mood and cognitive performance while inhibiting inflammation [3,37,38].

This study aims to develop and characterize microparticles encapsulating sage extract along with prebiotic compounds (inulin and pectin) to improve the delivery and bioavailability of sage’s bioactive constituents. The formulation strategy emphasizes the selection of suitable materials and techniques to ensure particle stability, functionality, and controlled release properties. While stability ensures the particle exists and controlled release dictates when the drug is liberated, functionality is the term for how well the particle performs its entire intended job: protecting, delivering, and enhancing the efficacy of its payload. Pectin, inulin, alginate, and sage extract are all natural food-grade ingredients with established safety profiles and are generally recognized as safe, their combined use in a concentrated microbead formulation warrants careful evaluation of potential toxicity. Encapsulation can alter the bioavailability, local concentration, and cellular interactions of the incorporated compounds, which may influence cytotoxicity, inflammatory responses, or other unintended effects. For example, high local concentrations of phenolic compounds or rapid release from damaged beads could theoretically affect epithelial cells or gut microbiota. Therefore, even when using well-tolerated food additives, it is essential to assess cytocompatibility, cell viability, and any pro-inflammatory effects in vitro as part of responsible formulation development.

Safety and efficacy evaluations are conducted using the Caco-2 intestinal epithelial cell line, a widely accepted in vitro model for investigating drug absorption and intestinal barrier function, by assessing biocompatibility, cytotoxicity, cellular uptake, and potential genotoxicity of the microparticles. In addition to these assessments, we investigated the impact of the formulation on the growth and viability of Lactobacillus strains, given the known prebiotic properties of inulin and pectin. This aspect aims to evaluate the potential of the microparticles to support beneficial gut microbiota, thereby enhancing the overall therapeutic benefits.

Despite extensive prior use of alginate, pectin, and inulin in encapsulation of bioactive compounds, the innovation of our work lies in the co-encapsulation of *Salvia officinalis* extract with prebiotic polysaccharides inulin and pectin within alginate microbeads, optimizing a multifunctional system that combines controlled release, enhanced probiotic support, and intestinal barrier protection. Encapsulation of sage extract is necessary because its bioactive compounds are inherently unstable and prone to degradation under physiological and environmental conditions. In their natural form, these molecules can be rapidly oxidized, hydrolyzed, or metabolized, which significantly reduces their bioavailability and therapeutic effectiveness. Moreover, direct application of free extract often results in poor solubility, uncontrolled release, and limited absorption across biological barriers such as the gastrointestinal epithelium.

Encapsulation into biopolymeric carriers as alginate, pectin, inulin protects the sensitive constituents from oxidation, light, pH fluctuations, and enzymatic degradation. At the same time, it allows for controlled and sustained release, enhances permeability, and improves bioavailability. This strategy also enables targeted delivery, reducing potential side effects and allowing lower effective doses. Another strength of this approach is the possibility of controlled and site-specific release: the hydrogel matrix responds to pH variations along the gastrointestinal tract, ensuring that the release of sage extract is sustained and occurs preferentially in the intestine [39,40]. One of the most important benefits is that it operates under mild, aqueous conditions without exposure to high temperatures or organic solvents, thereby preserving sensitive bioactive compounds like rosmarinic acid, carnosol, and carnosic acid [40]. In addition, polysaccharide-based ionic crosslinking typically achieves high encapsulation efficiencies, often exceeding 80–90%, whereas techniques such as solvent evaporation or emulsification may lead to partial loss of hydrophilic constituents [39,40]. Beyond their prebiotic properties, inulin and pectin were blended with alginate mainly to enhance the performance of the bead matrix. Both polysaccharides improve gel strength, stability, and encapsulation efficiency, while also modulating the porosity and swelling behavior of the beads. Pectin contributes additional resistance under acidic gastric conditions, helping to protect the encapsulated sage extract, whereas inulin can reduce bead brittleness and support a more sustained release profile. In this way, their inclusion complements alginate, resulting in a more robust and functional delivery system.

This integrative approach may not only improve the bioavailability and stability of the phytochemicals but also harness synergistic effects for gut health, which have been insufficiently explored in previous studies. Several studies have investigated the encapsulation of *Salvia officinalis* extracts and their main bioactive compounds. For instance, gelatin–pectin complex coacervation produced microcapsules with nearly 97% encapsulation efficiency and a particle size of approximately 100 µm, while effectively protecting phenolic constituents such as rosmarinic acid, chlorogenic acid, rutin, and quercitrin [41]. Other research focused on nanoencapsulation of sage leaf extract using whey protein isolate and seed gum coatings, which significantly improved the oxidative stability of sunflower oil compared to the free extract [42].

Encapsulation has also been applied to isolated active compounds. Carnosic acid was successfully incorporated into albumin, chitosan, and cellulose nanoparticles, resulting in enhanced biological activity in cell models such as MCF-7 and Caco-2 cells compared to the free compound. Similarly, recent studies using chitosan-based nanocapsules (90–150 nm) demonstrated long-term colloidal stability, reduced carrier cytotoxicity, and protection of neuronal cells in vitro [43].

Rosmarinic acid has also been encapsulated into PLGA nanoparticles via low-energy processes, which improved its stability and enabled controlled release, further supporting the potential of nanocarrier systems for sage-derived compounds [44]. Overall, these findings indicate that encapsulation not only protects sage phenolics from oxidation, light, and pH fluctuations but also allows for high encapsulation efficiencies, sustained release, and preserved or even enhanced bioactivity. The choice of matrix materials such as alginate, pectin, proteins, or chitosan enables tailoring of release kinetics and stability, while nanoscale formulations improve dispersion and cellular interactions. Collectively, these studies provide a strong basis for the development of functional delivery systems that maximize the therapeutic and nutraceutical potential of *S. officinalis* extracts.

Our research further explores the synergistic effects of sage extract combined with prebiotics on cellular health, leveraging their well-documented antioxidant, anti-inflammatory, and gut-modulatory activities. Ultimately, this work seeks to advance the development of effective, biocompatible microparticle-based delivery systems suitable for pharmaceutical and nutraceutical applications.

## 2. Materials and Methods

### 2.1. Materials

*Salvia officinalis* extract was obtained from Herbal Discont (Medinvest Hungary Ltd., Budapest, Hungary). Pectin was obtained from Cargill France SAS (Puteaus, France). The pectin (UniPECTINE^®^) used in the present study, obtained from Cargill France SAS (Puteaux, France), is a high-methoxyl (HM) pectin derived from citrus peels and apple pomace, with a degree of esterification of approximately 60%. It is water-soluble, and its gelation is optimal under acidic conditions (pH 3.1–3.8) and in the presence of high sugar concentrations. Inulin (Frutafit^®^) was obtained from SENSUS (Roosendaal, The Netherland) from chicory root and has an average degree of polymerization of around 9, corresponding to a molecular weight of approximately 5 kDa. It is soluble in water. Sodium alginate was obtained from BÜCHI Labortechnik AG (Flawil, Switzerland). It is extracted from brown seaweed (mannuronic to guluronic acid (M/G) ratio ranges from 1.5:1 to 2.5:1). The human adenocarcinoma cancer cell line (Caco-2) originated from the European Collection of Authenticated Cell Cultures (ECACC, Public Health England, Salisbury, UK). TrypLE™ Express Enzyme (no phenol red) was bought from Thermo Fisher Scientific (Waltham, MA, USA). The 96-well cell culture plates, and culturing flasks were purchased from VWR International (Debrecen, Hungary). Transwell^®^ 24-well cell culture inserts were supplied by Greiner Bio-One Hungary Kft. (Mosonmagyaróvár, Hungary). CUPRAC assay was obtained from G-Biosciences (St Louis, MO, USA). All other products were purchased from Sigma-Aldrich (St. Louis, MI, USA).

### 2.2. Formulation of Sage Loaded Alginate Beads

To synthesize alginate microparticles, 1 g of sodium alginate powder was dissolved in 100 mL of distilled water to prepare a 1% (*w*/*v*) alginate solution, considering the hygroscopic nature of sodium alginate. For the calcium crosslinking solution, 14.7 g of calcium chloride dihydrate was dissolved in 1 L of distilled water to obtain a 100 mM calcium chloride solution. Next, 5 g of *Salvia officinalis* extract (containing 55 µg/mg carnosol, Figure S1), 5 g of inulin, and 2 g of pectin were added to the alginate solution and stirred until fully dissolved. To comprehensively evaluate the formulations, both experimental and control microbeads were prepared. These included microbeads loaded with *Salvia officinalis* extract but without prebiotics, microbeads containing only inulin, microbeads containing only pectin, microbeads containing both inulin and pectin, and empty microbeads composed of alginate alone without extract or prebiotics.

The compositions of these microbeads were as follows: all formulations contained 100 mL of 1% alginate solution. The *S. officinalis* microbeads without prebiotics included 5 g of sage extract. The inulin-only microbeads contained 5 g of inulin, the pectin-only microbeads contained 2 g of pectin, and the combined inulin + pectin microbeads contained both 5 g of inulin and 2 g of pectin. Empty microbeads consisted solely of the alginate solution without any added extract or prebiotics.

The homogeneous mixtures were loaded into a syringe and processed using a BÜCHI B-395 Pro (BÜCHI Labortechnik AG, Flawil, Switzerland) encapsulation device. In this study, operational parameters were selected based on our previous experimental work and supporting literature data, as these have consistently shown that nozzle diameter, vibration frequency, flow rate, and electrostatic voltage are critical determinants of microsphere size uniformity and encapsulation efficiency [45,46,47]. Specifically, a nozzle diameter of 200 μm, vibration frequency of 1600 Hz, electrostatic voltage ranging from 1000 to 1200 V, and flow rate of 5.36 mL/min were applied using the encapsulator. A large, flat-bottomed beaker containing the 100 mM calcium chloride solution was placed beneath the nozzle on a magnetic stirrer. The formed alginate beads were allowed to cure in the calcium chloride bath for 15 min to ensure proper gelation. Following this, the microparticles were rinsed with distilled water, collected by vacuum filtration through a membrane with a pore size of 0.4 µm, and were subsequently freeze-dried using a Scanvac CoolSafe Touch 110-4 (LaboGene ApS, Allerød, Denmark) freeze dryer at −110 °C for 24 h.

### 2.3. Scanning Electron Microscopy Analysis

For morphological characterization, scanning electron microscopy (SEM) was carried out using a Thermo Scientific™ Axia™ ChemiSEM™ Scanning Electron Microscope (Auro-Science Consulting, Budapest, Hungary). The samples were attached to a fixture with a double-sided adhesive tape containing graphite and the excess amount was washed off with argon gas. Any surface pre-treatments and ex-post corrections not used during the measurement. The measurement requires a high vacuum, 30 kV accelerating voltage, and 15 µs dwell time. The magnifications were 1000× in all cases.

### 2.4. Encapsulation Efficiency

The efficiency of drug entrapment within the microparticles was evaluated by sampling 1 mL of the calcium chloride (100 mM) hardening solution immediately after bead formation. The concentration of unencapsulated compound was quantified using a UV-VIS spectrophotometer at a wavelength of 285 nm, which corresponds to the absorption maximum of carnosol [20]. The amount of active agent retained within the microparticles—specifically, carnosol from *S. officinalis*—was calculated based on the difference between the initial drug amount and the free drug concentration measured in the hardening bath. The encapsulation efficiency (EE%) was determined using the following equation (Equation (1)):

(1) $$Encapsulation\;efficiency\left(EE\%\right)=\frac{amount\;of\;initial\;drug-amount\;of\;free\left(not\;formulated\right)\;drug\;(mg)}{amount\;of\;initial\;drug\;(mg)}\times 100$$

### 2.5. Swelling Behavior

The hydration properties of various formulations were assessed by swelling analysis. To evaluate the water uptake capacity, 50 mg samples of different microparticles were immersed in 50 mL of purified water. The tested formulations included beads containing only inulin, only pectin, both inulin and pectin, a combination of inulin, pectin, and *S. officinalis* extract, as well as empty microbeads without any active components. The suspensions were stirred at 37 °C using a Radelkis OP-912 magnetic stirrer (Radelkis, Budapest, Hungary).

After 24 h of incubation, the beads were carefully collected, gently blotted with filter paper to remove surface moisture, and immediately weighed. The equilibrium water uptake (EWU) was expressed as a percentage and calculated using the following formula (Equation (2)):

(2) $$EWU=\left(\frac{Ws-Wd}{Ws}\right)\times 100$$

where Ws represents the weight of the hydrated particles and Wd is the initial dry weight, as described by Manjanna et al. (2010) [47].

### 2.6. In Vitro Release Profile

The in vitro drug release of carnosol from the formulated microbeads and from the sage extract was performed using USP dissolution apparatus (Erweka, DT 800, Erweka Gmbh, Heusenstamm, Germany). The simulated intestinal fluid (SIF) used in this study was prepared according to the USP and European Pharmacopoeia guidelines for enzyme-free SIF. It consisted 13.872 g of potassium dihydrogen phosphate (KH_2_PO_4_) anf 35.084 g of disodium hydrogen phosphate (Na_2_HPO_4_) in sufficient water to produce 1000 mL [48]. The dissolution medium consisted of 900 mL of freshly prepared SIF without pancreatin (pH 6.8) maintained at 37 °C; the paddle speed was set at 100 rpm. From the different formulations, an amount equivalent to 20 mg of the *S. officinalis* was filled in a hydroxypropyl methylcellulose (HPMC) (Capsugel, Inc., Morristown, NJ, USA) capsule size “0”. As a control, capsules were filled with non-microencapsulated lyophilized sage extract. Sampling was performed at strategically selected time points (0, 30, 60, 120 min) with 1 mL aliquots withdrawn through a 0.45 µm syringe filter to ensure particulate-free analysis. Carnosol release was quantified via UV-Vis spectrophotometric measurements at 285 nm wavelength [20]. All concentration determinations were referenced against a pre-established carnosol calibration curve. The calibration curve was linear, with an R^2^ value of 0.995, and the concentration range was between 1 and 100 µg/mL.

### 2.7. Assessment of Enzymatic Stability

To evaluate the resistance of the microbeads to enzymatic degradation, experiments were carried out in simulated gastric fluid (SGF) containing pepsin and simulated intestinal fluid (SIF) containing pancreatin, prepared in accordance with European Pharmacopoeia guidelines [48]. The pepsin used had an activity of approximately 400–450 units/mg solid, while the pancreatin exhibited a protease activity of about 203 USP units/mg. Samples were placed into 100 mL of SGF for 2 h and into SIF for 2 h. Incubations were performed at 37 °C with continuous agitation at 100 rpm. At selected intervals over a 120 min period, 1 mL samples were withdrawn and promptly mixed with an equal volume of ice-cold quenching reagent—0.10 M NaOH for SGF or 0.10 M HCl for SIF—to halt enzymatic activity. The amount of carnosol remaining within the microbeads after exposure to the enzymatic degradation was subsequently quantified spectrophotometrically at 285 nm.

### 2.8. Caco-2 Cell Line

For the MTT and permeability evaluations, the Caco-2 cell line—derived from human colorectal adenocarcinoma and known for its immortalized phenotype—was utilized, as referenced in [49]. The cells were cultured in standard plastic flasks and were routinely subcultured on a weekly basis. They were grown in Dulbecco’s Modified Eagle Medium (DMEM), enriched with 2 mM l-glutamine, 100 mg/L gentamicin, and 10% fetal bovine serum that had been heat-inactivated. All cultures were maintained in a humidified incubator set to 37 °C with an atmosphere containing 5% CO_2_.

### 2.9. MTT Viability Assay

The metabolic activity of Caco-2 cells was assessed using the MTT assay to determine cellular viability. Cells were seeded in 96-well plates at a density of 10^4^ cells per well in Dulbecco’s Modified Eagle Medium (DMEM) and cultured until full confluence was reached. The samples were prepared by suspending either the microbeads or the free sage extract in PBS. The suspensions were homogenized using a vortex mixer and subsequently sonicated to ensure uniform dispersion. Finally, the suspensions were filtered through a 0.45 µm membrane to completely remove polymer particles and any potential microbial contaminants, yielding a sterile solution containing the soluble compounds available for cellular application.

After confluency, the culture medium was removed and cells were treated with various concentrations of either free *S. officinalis* extract or *S. officinalis*-loaded microbeads, diluted in culture medium, and incubated for 24 h at 37 °C in a humidified atmosphere containing 5% CO_2_. After treatment, the microbeads were removed, and 100 µL of a 0.5 mg/mL MTT solution was added to each well. Plates were incubated for an additional 3 h in the dark at 37 °C. Subsequently, the supernatant was discarded, and the formazan crystals formed by mitochondrial reduction of MTT were solubilized using a 25:1 mixture of 2-propanol and hydrochloric acid. The reduction of yellow tetrazolium salt to purple, water-insoluble formazan served as an indicator of metabolic activity. Absorbance was measured at 570 nm using a Multiskan GO microplate reader (Thermo Fisher Scientific, Waltham, MA, USA). Cell viability was calculated as a percentage relative to untreated control cells (PBS-treated), which were considered 100% viable. Triton X-100 (10%) was used as a positive control to induce cytotoxicity. All experiments were conducted in triplicate, and data are expressed as mean ± standard deviation.

### 2.10. Transepithelial Electrical Resistance (TEER) Evaluation

To assess the integrity of the epithelial barrier formed by Caco-2 cells, transepithelial electrical resistance (TEER) measurements were performed. Cells were seeded at a density of 4 × 10^4^ cells per well and cultured until they formed tight monolayers. Electrical resistance was measured using the Millicell-ERS system (Millipore, Merck, Waltham, MA, USA), and only monolayers with TEER values between 800 and 1000 Ω·cm^2^ were selected for further experiments.

Test formulations were prepared by suspending microbeads or free sage extract in PBS at concentrations of 1% (*w*/*v*) for microbeads and equivalent extract mass for the free extract. The suspensions were homogenized by vortexing and sonicated to ensure uniform dispersion. The suspensions were filtered through a 0.45 µm membrane to completely remove polymer particles and any potential microbial contaminants, yielding a sterile solution containing the soluble compounds available for cellular application. During the assay, cells were treated with these test formulations, and TEER values were continuously recorded for 1 h to monitor the immediate effects on barrier integrity.

Phosphate-buffered saline (PBS) and 10% (*w*/*v*) Triton X-100 (Sigma-Aldrich (St. Louis, MI, USA) served as integrity-preserving and barrier-disruptive controls, respectively. To evaluate barrier recovery, TEER monitoring was extended for an additional 12 h post-treatment to assess the restoration of tight junction functionality [50].

### 2.11. In Vitro Permeability Assay

Permeability assessments were conducted using Caco-2 cell monolayers cultured on Transwell^®^ inserts (24-well format, polycarbonate membrane, surface area 1.12 cm^2^, pore size 0.4 µm). Cells were seeded at a density of 4 × 10^4^ cells per insert, and monolayer integrity was confirmed by measuring transepithelial electrical resistance (TEER) prior to the assay.

For the permeability test, the same test samples were used as in the TEER evaluation (1% (*w*/*v*) for microbeads and equivalent extract mass for the free extract in PBS). Before application, the dispersions were mixed well to maintain homogeneity. A volume of 400 µL of this mixture was applied to the apical compartment of each insert, while 1400 µL of culture medium was added to the basolateral compartment. Aliquots of 50 µL were withdrawn from the basolateral side at 2, 4, and 24 h to monitor compound permeation.

The samples collected were analyzed by UV-Vis spectrophotometry at 285 nm to determine the amount of carnosol permeated through the Caco-2 cell monolayer.

### 2.12. DPPH Antioxidant Test

The antioxidant potential of the formulations was assessed using the DPPH assay, a colorimetric method based on the reduction of 2,2-diphenyl-1-picrylhydrazyl (DPPH). In the presence of antioxidant agents, the stable free radical DPPH undergoes a visible color change from deep violet to yellow, indicating its neutralization. For the assay, a 0.06 mM DPPH solution was prepared by dissolving DPPH powder (molecular weight: 394.33 g/mol) in 96% ethanol. Samples were prepared by suspending 100 mg of microbeads and free sage extract in 100 mL of PBS at a concentration of 10% (*w*/*v*). After that the suspensions were filtered through a 0.45 µm membrane to completely remove polymer particles and any potential microbial contaminants, yielding a sterile solution containing the soluble compounds available for cellular application.

Each test consisted of mixing 100 µL of the PBS-diluted sample with 2 mL of the DPPH solution. The mixtures were incubated in the dark at room temperature for 30 min to prevent light-induced degradation. Trolox (10.0 µM in PBS) was used as a reference antioxidant, while the DPPH solution without sample served as the negative control. The remaining concentration of DPPH was quantified spectrophotometrically by measuring absorbance at 517 nm. The antioxidant activity (AA%) of each formulation was calculated using the standard formula described in the literature [51] (Equation (3)):

(3) AA% = [(Abs control − Abs sample)/Abs control] × 100

where Abs control is the absorbance of the DPPH solution without the sample and Abs sample is the absorbance of the sample after it has reacted with the DPPH radical.

### 2.13. CUPric Reducing Antioxidant Capacity Assay (CUPRAC)

The CUPRAC assay can be used to assess an antioxidant’s ability to inhibit reactive oxygen species (ROS) by measuring the antioxidant’s capacity to reduce Cu(II) to Cu(I) in the presence of ROS.

For the CUPRAC assay, the reagents were prepared according to the manufacturer’s instructions. The samples were prepared by suspending either the microbeads or the free sage extract in PBS. The suspensions were homogenized by using vortex. The suspensions were filtered through a 0.45 µm membrane to completely remove polymer particles and any potential microbial contaminants.

Samples and Trolox standards were pipetted into the wells of a 96-well microplate, and the working reagent was subsequently added, ensuring the final composition. The plate was incubated at room temperature for 30 min to allow the reaction to proceed.

After incubation, absorbance was measured at 450 nm using a UV-VIS spectrophotometer. A calibration curve was established using Trolox solutions prepared from a 10 µM stock solution. The absorbance values of the standards were plotted against concentration, and the antioxidant capacities of the samples were calculated from the regression equation. Results are expressed as micromolar Trolox equivalents, and all measurements were carried out in triplicate.

### 2.14. Anti-Inflammatory Assay

The anti-inflammatory potential of the formulations was investigated in vitro using ELISA assays targeting human TNF-α (Sigma-RAB0476) and IL-1β (Sigma-RAB0273) (Sigma-Aldrich (St. Louis, MI, USA) in Caco-2 epithelial cells seeded at 10^4^ cells per well in 96-well plates. Test samples were prepared by suspending 100 mg of each formulation in phosphate-buffered saline (PBS) to reach a final concentration of 1% (*w*/*v*).

Two experimental setups were employed. In the first, inflammation was induced by treating cells with 1 mM hydrogen peroxide (H_2_O_2_) for 4 h, followed by replacement of the medium with fresh test solution and further incubation for 20 h to assess therapeutic effects. In the second setup, cells were simultaneously exposed to 1 mM H_2_O_2_ and the test formulation for 24 h, modeling concurrent inflammation and treatment to evaluate potential prophylactic effects.

All incubations were conducted at 37 °C in a humidified atmosphere with 5% CO_2_. After the incubation periods, supernatants were collected, and TNF-α and IL-1β levels were measured via ELISA according to the manufacturer’s instructions.

### 2.15. Growth Curve Determination for Lactobacillus Strains

The bacterial strains *Lactobacillus plantarum* (45210), *Lactobacillus rhamnosus* (Br-p1), and *Lactobacillus brevis* (Pr-p2) were obtained from the strain collection of the Metagenomics Institute (University of Debrecen). Strain 45210 was originally isolated from human fecal material, while Br-p1 and Pr-p2 were isolated from broccoli and red currant, respectively. All strains were cultivated in de Man, Rogosa, and Sharpe (MRS) medium at 37 °C under aerobic conditions. Prior to inoculation, the bacterial strains were pre-cultured on solid MRS agar plates to ensure both viability and purity. Bacterial suspensions were prepared from 24 h cultures that had been grown on MRS agar. Each suspension was adjusted to 0.5 McFarland standard, corresponding to approximately 1.5 × 10^8^ CFU/mL.

To evaluate the effect of our preparation on bacterial growth, we added our formulation to MRS medium at final concentrations of 2.5%, 5%, and 7.5% (*w*/*v*). A control group without our formulation was also included in the study. All treatments were prepared in at least two independent replicates. The bacterial growth curve was monitored in liquid MRS medium under each experimental condition. Cultures were subjected to static incubation at 37 °C under anaerobic conditions for a total duration of 10 days. The number of viable cells was determined by taking samples on days 0, 1, 2, 5, 8 and 10.

At each designated time point, 100 µL aliquots of the cultures were serially diluted with sterile saline (0.9% *m*/*m*) and plated on MRS agar in duplicate. Following a 48 h incubation at 37 °C under aerobic conditions, the colony-forming units (CFUs) were counted. Image analysis for colony enumeration was conducted utilizing Fiji software (ImageJ version 1.54p, NIH), thereby facilitating objective and standardized quantification. All experimental procedures, including media preparation, inoculation, and sampling, were conducted under aseptic conditions using a laminar flow cabinet to minimize the risk of contamination.

### 2.16. Statistical Analysis

All quantitative results are presented as mean values ± standard deviations (SDs), calculated from minimum three independent replicates. The dataset—including results from swelling analysis, in vitro release testing, enzymatic degradation, permeability studies, TEER evaluation, DPPH antioxidant activity, anti-inflammatory efficacy, and MTT-based viability screening—was analyzed using GraphPad Prism software (version 10.4; GraphPad Software, San Diego, CA, USA). One-way and repeated measures ANOVA were employed to assess statistical significance, followed by appropriate post hoc tests (Tukey’s or Dunnett’s) for group comparisons. Statistical significance was defined as *p* < 0.05.

## 3. Results

### 3.1. Morphology

**Figure 1:**
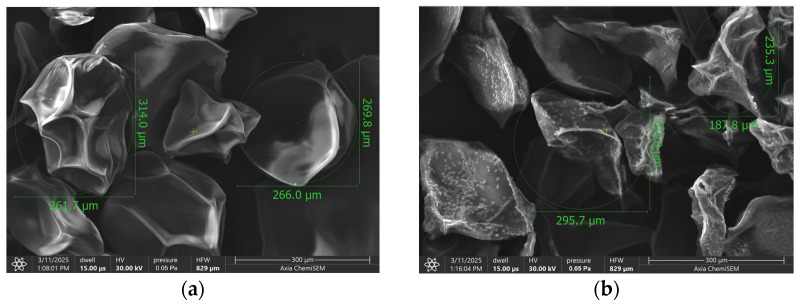
SEM image of (**a**) empty microbeads and (**b**) *S. officinalis*- and prebiotics-loaded microbeads.

The morphology of the lyophilized Salvia-loaded alginate microparticles is shown in Figure 1. The beads exhibited irregular, somewhat angular shapes with clear, faceted surfaces. Particle diameters ranged from approximately 235 µm to 314 µm, with empty beads having a mean diameter of 273 ± 12.5 µm and *Salvia officinalis* loaded beads measuring 275 ± 14.03 µm. The surface texture appeared wrinkled and folded, with some areas displaying pronounced ridges and valleys. The particles were well separated, showing minimal visible agglomeration at this magnification. Additionally, small calcium chloride crystals were observed on the bead surfaces. (Figure S2) Their presence on the loaded beads can be explained by differences in surface chemistry and local microenvironments during gelation and drying. In sage-loaded beads, the presence of concentrated extract components and the polysaccharide–extract interactions can create localized zones of higher ionic concentration, which promote crystallization of calcium chloride on the surface. In contrast, empty beads, composed only of the alginate–pectin–inulin matrix, present a more homogeneous surface and ion distribution, preventing the formation of visible calcium chloride crystals under the same crosslinking and drying conditions.

### 3.2. Encapsulation Efficiency

The encapsulation efficiency (EE) of the multifunctional microbeads—incorporating *S. officinalis* extract, inulin, and pectin within an alginate matrix—demonstrated exceptional performance, consistently exceeding 90% across all experimental replicates, with *S. officinalis*-loaded beads achieving an average encapsulation efficiency of 94 ± 0.57%. This remarkably high retention rate was verified through spectrophotometric analysis of the crosslinking medium.

### 3.3. Swelling Behavior

**Figure 2:**
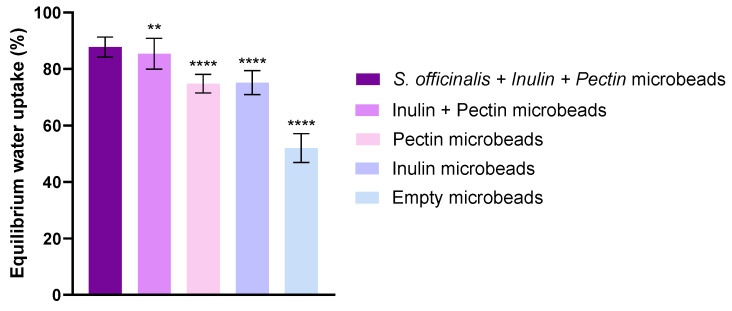
Swelling capacity of the beads in distilled water. Values represent equilibrium water uptake for each composition (mean ± SD, n = 5). Statistical analysis was performed using one-way ANOVA with Dunnett’s multiple comparison test. Statistically significant differences are indicated by ** (*p* < 0.01) and **** (*p* < 0.0001).

The equilibrium water uptake for every group was calculated as described. Figure 2 shows the percentage increase in bead weight due to water absorption after 24 h. Each bead types exhibited high equilibrium water uptake. The microbeads containing *S. officinalis*, inulin and pectin (*S. officinalis* microbeads) demonstrated higher water uptake, approximately 88%. The water uptake of microbeads prepared with the combination of inulin + pectin but without extract was slightly lower, ~85%, but still significantly higher than that of the single-component formulations. Formulation containing only pectin achieved a water uptake of approximately 78%, while inulin-based spheres showed a slightly lower value at 72%. Empty microbeads showed the lowest water uptake, which was significantly different from the other formulations. The incorporation of *Salvia* extract, inulin, and pectin into the beads increased their equilibrium water uptake relative to the empty microbeads. This suggests that the presence of these components enhances the hydrophilicity or porosity of the bead matrix, allowing greater water absorption at equilibrium.

### 3.4. In Vitro Release Profil

**Figure 3:**
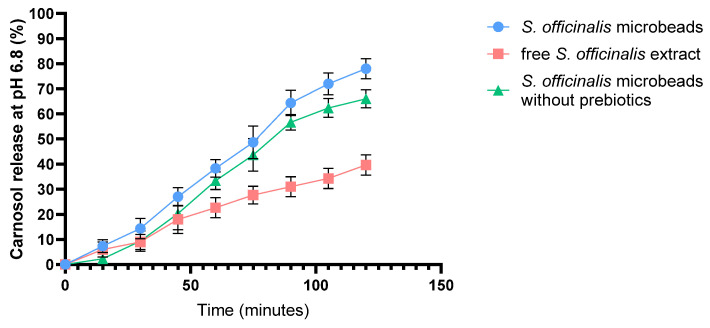
In vitro dissolution profile of carnosol from *S. officinalis* microbeads, free *S. officinalis* extract, and *S. officinalis* microbeads without prebiotics in simulated intestinal fluid (SIF) without pancreatin (pH = 6.8). Each data point represents the mean ± SD; n = 3.

Figure 3 presents the dissolution profile of carnosol over 2 h in SIF comparing an alginate bead formulation containing *S. officinalis* extract, inulin, and pectin, and *S. officinalis* microbeads without prebiotics with a control consisting of non-encapsulated (free) *S. officinalis* extract. As shown in the graph, the *S. officinalis* microbeads (blue line) *(S. officinalis*, inulin and pectin) exhibited a significantly higher and more sustained release of carnosol compared to the non-encapsulated *S. officinalis* extract (red line) throughout the 120 min observation period. The cumulative release from the *S. officinalis* microbeads increased steadily, reaching approximately 85% at the end of the experiment. Microbeads without prebiotics (green line) showed a similar, slightly lower release of around 80%. In contrast, the free extract alone was markedly lower, not exceeding 45%.

These results indicate that encapsulating sage extract with prebiotic compounds significantly enhances the release profile of carnosol under simulated intestinal conditions (pH 6.8), likely providing improved bioavailability compared to non-encapsulated extract. Error bars denote standard deviations from triplicate measurements.

### 3.5. Enzymatic Stability Assay

**Figure 4:**
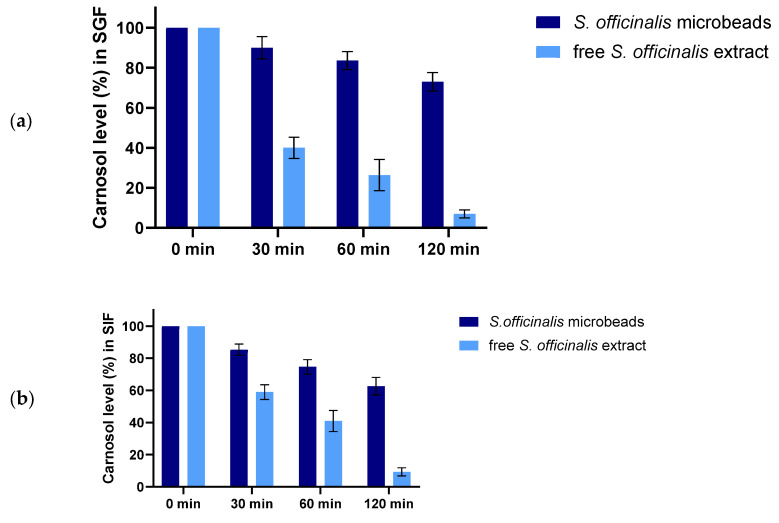
Enzymatic stability of microbeads and free *S. officinalis* extract in SGF (**a**) and in SIF medium (**b**). Each data point represents the mean ± SD; n = 3.

Figure 4 shows the enzymatic degradation profiles of *S. officinalis* microbeads and free *S. officinalis* extract, under (a) simulated gastric fluid (SGF, pH1.2) and (b) simulated intestinal fluid (SIF, pH6.8) conditions.

In SGF (a), the carnosol content of free *Salvia* extract drops rapidly, with less than 10% remaining after 120 min. In contrast, the encapsulated *Salvia* (prebiotic beads) retains a significantly higher percentage of carnosol over the same period, indicating enhanced protection against acidic, enzymatic degradation. The bead-matrix effectively shields carnosol, maintaining over 60% of its initial content at 120 min.

In SIF (b), both samples experience a gradual decrease in carnosol content. However, the prebiotic beads again demonstrate greater stability, with approximately 60% of carnosol remaining after 2 h, while the free extract drops below 20%. These findings confirm that encapsulation within alginate microbeads confers substantial protection for carnosol under both gastric and intestinal conditions, thereby improving its stability and potential bioavailability throughout gastrointestinal transit.

### 3.6. MTT Viability Assay

**Figure 5:**
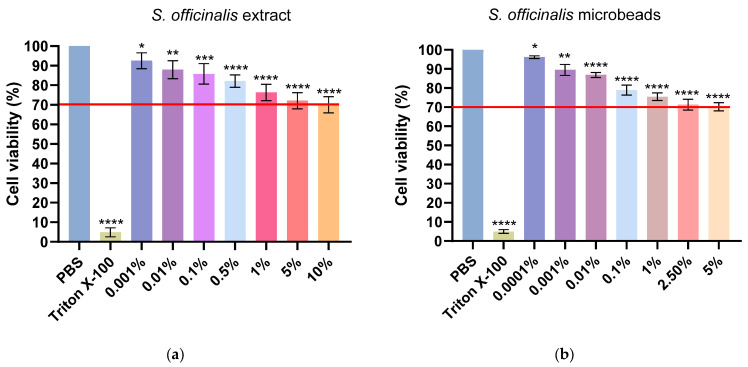
Viability of Caco-2 cells incubated with free *S. officinalis* extract (**a**) and *S. officinalis* microbeads (**b**). Viability is expressed as a percentage of the negative control (PBS). Data are presented as mean ± SD; n = 6. Ordinary one-way ANOVA with Dunnett’s multiple comparison test was performed to compare the different formulations with PBS. Statistically significant differences are indicated by *, **, ***, and **** for *p* < 0.05, *p* < 0.01, *p* < 0.001 and *p* < 0.0001, respectively.

Figure 5 displays the results of the MTT assay on Caco-2 cells, evaluating cell viability following exposure to varying concentrations of free *Salvia* extract (Figure 5a) and *Salvia*-loaded microbeads Figure 5b. In both experiments, phosphate-buffered saline (PBS) serves as the negative control (indicating maximal cell viability), while Triton X-100 represents the positive (cytotoxic) control, resulting in minimal cell viability. The red horizontal line marks the 70% cell viability threshold, commonly considered the cytotoxicity cut-off.

In case of the results, corresponding to the free *Salvia* extract, cell viability remains above 80% at lower concentrations (0.001–0.1%), but gradually decreases as the concentration increases, falling slightly below the 70% threshold at concentrations of 1% and higher. This suggests that higher concentrations of the free extract exhibit moderate cytotoxicity. As for the microbead formulation, the trend is similar: cell viability is highest at the lowest concentrations (0.0001–0.01%), staying well above the cytotoxicity threshold, but decreases to just under 70% as the concentration rises to 1% and higher. Notably, the microbead formulation consistently provides slightly better cell viability at comparable concentrations relative to the free extract. These data demonstrate that both formulations are relatively safe at lower concentrations, but higher concentrations can decrease cell viability below the accepted safety threshold. The microbead formulation exhibits marginally improved biocompatibility compared to the free extract, particularly at higher concentrations. Statistical significance is indicated above each bar, highlighting differences versus the control group. The red line represents the threshold for cytotoxicity, set at 70% cell viability compared to the negative control.

### 3.7. Transepithelial Electrical Resistance Measurements

**Figure 6:**
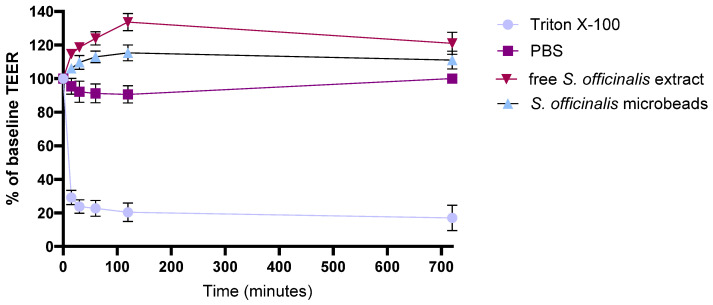
TEER assessment of Caco-2 cell barrier integrity after treatment with free *S. officinalis* extract and *S. officinalis* containing microbeads. Each data point represents the mean ± SD; n = 6.

The integrity of adenocarcinoma cell monolayers was quantitatively assessed by measuring transepithelial electrical resistance (TEER) over a 12 h period, with values reported as a percentage of baseline (Figure 6). Treatment with 10% (*w*/*v*) Triton X-100, used as a positive control, induced a rapid and pronounced reduction in TEER, confirming a complete loss of monolayer integrity. In contrast, PBS-treated monolayers (negative control) maintained TEER values consistently close to baseline throughout the experiment.

Upon treatment with 1% (*w*/*v*) free *S. officinalis* extract, TEER values gradually increased to around 120% of the initial value and remained elevated across all measured time points, suggesting that components in the extract may reinforce or tighten the intercellular junctions. A comparable, slightly more pronounced effect was observed with microbeads containing *S. officinalis*, inulin, and pectin, where TEER values not only rose above baseline but also stabilized at higher levels than those observed with the extract alone, likely due to the synergistic contributions of the prebiotic ingredients.

Overall, these results demonstrate that both the *S. officinalis* extract and the microbeads containing *S. officinalis*, inulin, and pectin contribute to enhanced epithelial barrier integrity, underscoring their potential for supporting intestinal barrier function and providing anti-inflammatory benefits.

### 3.8. In Vitro Permeability Assay

**Figure 7:**
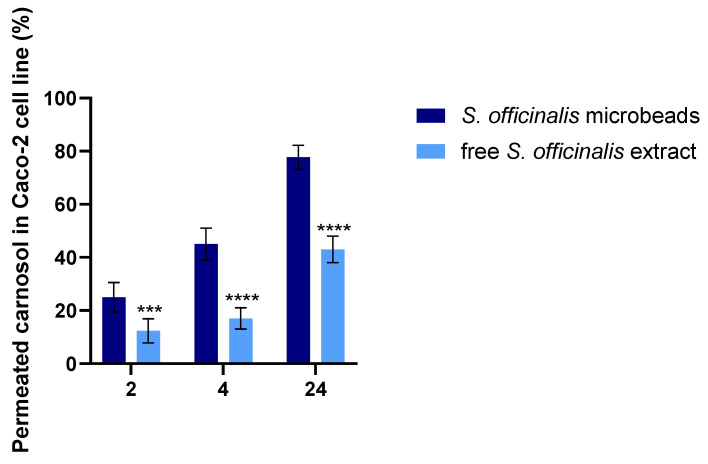
In vitro permeation of carnosol through Caco-2 cells from *S. officinalis* microbeads and free *S. officinalis* extract. Statistical analysis was performed using one-way ANOVA followed by Tukey’s post hoc test to compare differences among treatment groups at each time point. A *p*-value of less than 0.05 was considered statistically significant. Data are presented as mean ± standard deviation. ***, and **** for *p* < 0.001 and *p* < 0.0001, respectively.

The permeability of carnosol across the Caco-2 monolayer was assessed when TEER values reached 800–1000 Ω·cm^2^, indicating an intact epithelial barrier. As illustrated in Figure 7, the cumulative percentage of carnosol that permeated to the basolateral compartment was measured at 2, 4, and 24 h for both non-encapsulated *Salvia* extract and *Salvia* prebiotic microbeads.

The results demonstrate that the microbeads achieved substantially greater carnosol permeability at all measured time points compared with the non-encapsulated sage extract. By 24 h, the percentage of permeated carnosol from the microbeads was approximately double that of the free extract, indicating superior intestinal absorption potential for the microencapsulated formulation.

### 3.9. DPPH Antioxidant Test

The radical scavenging capacity of the developed formulations was quantitatively assessed through the DPPH assay, with results expressed as percentage antioxidant activity (AA%). In this experimental setup, phosphate-buffered saline (PBS) served as the negative control baseline, whereas Trolox solution—a water-soluble vitamin E analog—demonstrated maximal reactive oxygen species (ROS) neutralization as the positive control.

**Figure 8:**
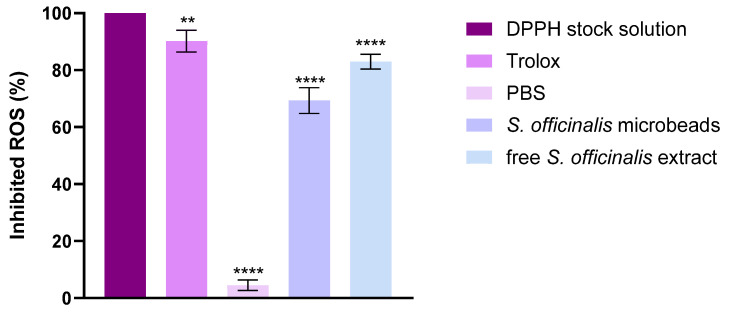
DPPH-scavenging activity of the composition. Data are presented as mean ± SD; n = 8. An ordinary one-way ANOVA with Dunett’s multiple comparison test was performed to compare the different formulations with PBS: ** and **** indicate statistically significant differences at *p* < 0.01 and *p* < 0.0001.

As illustrated in Figure 8, the tested botanical extracts displayed significant free radical inhibition potential. Notably, *Salvia* species extracts manifested particularly robust antioxidant performance, with *Salvia* prebiotic achieving 65% DPPH scavenging capacity. This substantial activity level positions the extract as a promising natural antioxidant source, comparable to many established bioactive compounds.

### 3.10. CUPric Reducing Antioxidant Capacity Assay (CUPRAC)

**Figure 9:**
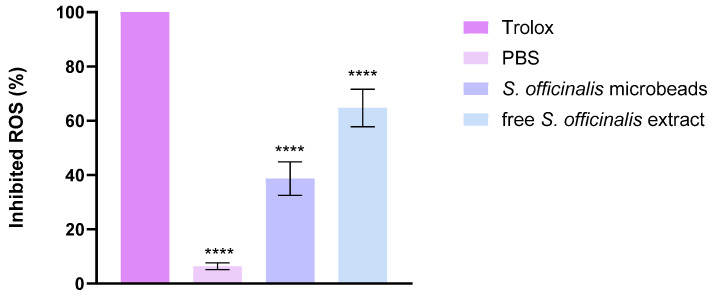
CUPric Reducing Antioxidant Capacity activity of the composition. Data are presented as mean ± SD; n = 3. An ordinary one-way ANOVA with Dunett’s multiple comparison test was performed to compare the different formulations with Trolox. **** indicate statistically significant differences at *p* < 0.0001.

The antioxidant activity of the investigated samples was evaluated using the CUPRAC assay, and the results are expressed as the percentage of inhibited ROS. Trolox, used as a positive control, showed almost complete ROS inhibition, confirming the reliability of the method. In contrast, PBS, serving as the negative control, exhibited only negligible antioxidant activity. As shown in Figure 9 both the *S. officinalis* microbeads and the free *S. officinalis* extract demonstrated significant ROS scavenging capacity compared to PBS. The microbeads inhibited approximately 35–40% of ROS, while the free extract achieved a higher effect of about 65–70%. Statistical analysis indicated highly significant differences (****, *p* < 0.0001) between the treatments and the control. These results indicate that *Salvia officinalis* possesses strong antioxidant activity in both encapsulated and free forms, although the free extract is more effective than the microbead formulation.

### 3.11. Anti-Inflammatory Assay

**Figure 10:**
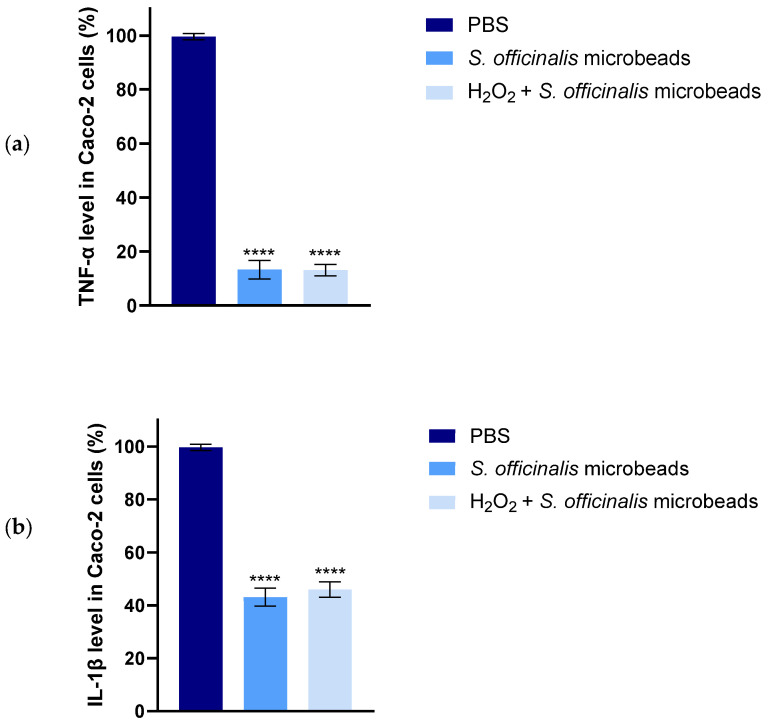
Results of human TNF-α (**a**) and IL-1β (**b**) ELISA tests on Caco-2 cells. Data are presented as mean ± SD; n = 6. Ordinary one-way ANOVA with Dunnett’s multiple comparison test was performed to compare the different formulations with PBS. The **** indicates statistically significant differences at *p* < 0.0001.

Based on the ELISA results for TNF-α and IL-1β cytokines, both inflammatory markers showed a significant decrease as shown in Figure 10. Figure 10a shows the percentage level of TNF-α in Caco-2 cells, and Figure 10b shows the percentage level of IL-1β in Caco-2 cells. The therapeutic potential of the formulations was more evident when applied after the inflammatory stimulus (post-treatment protocol). In this case, a significant decrease in both TNF-α and IL-1β secretion was observed in cells pretreated with 1 mM H_2_O_2_ for 4 h and then treated with the test formulations for 20 h. In the co-treatment setting, where the test formulations were administered together with H_2_O_2_ for 24 h, a moderate decrease in inflammatory cytokine levels was observed compared to the inflammatory control. The degree of inhibition was nearly identical to that achieved with the post-treatment protocol, suggesting that the preparations are equally effective in dampening an already established inflammatory response and preventing its development.

### 3.12. Growth Curve Determination for Lactobacillus Strains

**Figure 11:**
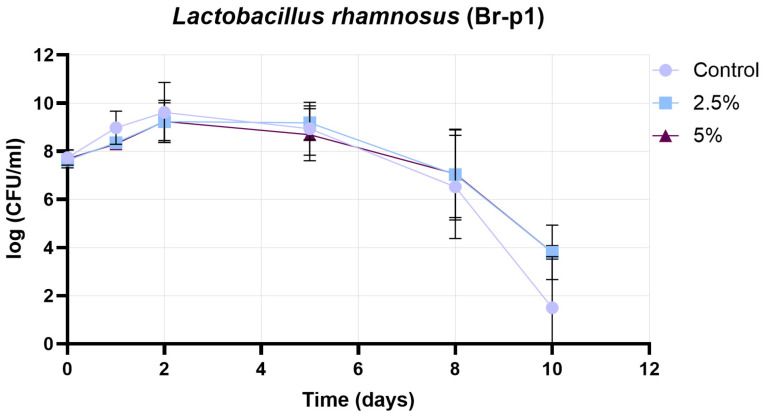
Results of growth curve determination for Br-p1 strain (*Lactobacillus rhamnosus*). Data are presented as mean ± SD; n = 3.

Figure 11 presents the growth curve of the Br-p1 strain (*Lactobacillus rhamnosus*), in which the change in cell count (log CFU/mL) is plotted as a function of time (days) under three conditions: control, 2.5% treatment, and 5% treatment. The initial cell count was found to be similar in all three cases (approximately 7.7 log CFU/mL). During the initial growth phase (days 0–1), the peak population was reached more rapidly in the control sample, while a more moderate growth was observed in the 2.5% and 5% treatments. The maximum cell count in the control group was recorded on day 2 (approximately 10 log CFU/mL), whereas in the 2.5% and 5% treatments, the peak was shifted to day 3 with slightly lower values (2.5%: approximately 9.8–10 log CFU/mL; 5%: approximately 9.5 log CFU/mL). During the stationary phase (days 3–5), a slow decrease in cell count was observed in all groups. In the 2.5% treatment, consistently higher values were maintained compared to the control, while in the 5% treatment group, the decrease occurred slightly faster but still remained above the control values. In the decline phase (days 6–10), the cell count in the control sample decreased rapidly, reaching practically zero by day 10. In contrast, a slower decrease was observed in the 2.5% and 5% treatments, with measurable cell counts still present on day 10 (approximately 3.8–4 log CFU/mL). Based on these results, no significant inhibition of Br-P1 growth was caused by the 2.5% and 5% treatments, while cell survival during the experimental period was improved. The most favorable balance between achieving the highest cell count and maintaining long-term viability was demonstrated by the 2.5% treatment.

**Figure 12:**
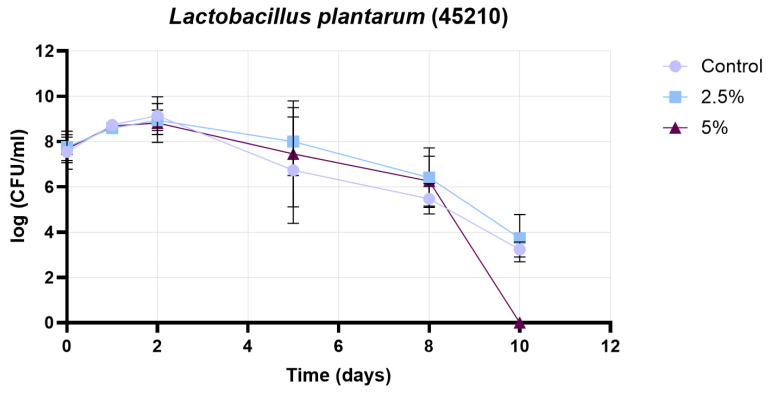
Results of growth curve determination for 45210 strain (*Lactobacillus plantarum*). Data are presented as mean ± SD; n = 3.

Figure 12 presents the growth curve of the 45210 strain (*Lactobacillus plantarum*), under three conditions: control, 2.5% treatment, and 5% treatment. The initial cell counts were comparable in all cases (approximately 7.7 log CFU/mL). During the early growth phase (days 0–2), all groups exhibited an increase in cell count, with the control reaching its maximum on day 2 (~9.3 log CFU/mL), while both the 2.5% and 5% treatments peaked at slightly lower levels (~8.9–9.0 log CFU/mL). Following the peak, a gradual decline in cell count was observed in all treatments. Between days 2 and 5, the 2.5% and 5% treatments maintained higher cell counts than the control, indicating a potential protective effect. From day 5 onwards, the control group experienced a faster decline, reaching ~3.4 log CFU/mL by day 10. In contrast, the 5% treatment showed a sharp drop after day 8, reaching zero by day 10, whereas the 2.5% treatment preserved a measurable cell population (~3.8 log CFU/mL) at the end of the experiment. These results suggest that, while both treatments influenced the growth dynamics of the 45210 strain, the 2.5% treatment provided a more consistent protective effect, supporting higher viability during prolonged incubation. In contrast, the 5% treatment initially maintained higher counts but ultimately led to a more pronounced decline in cell viability by the end of the study period.

**Figure 13:**
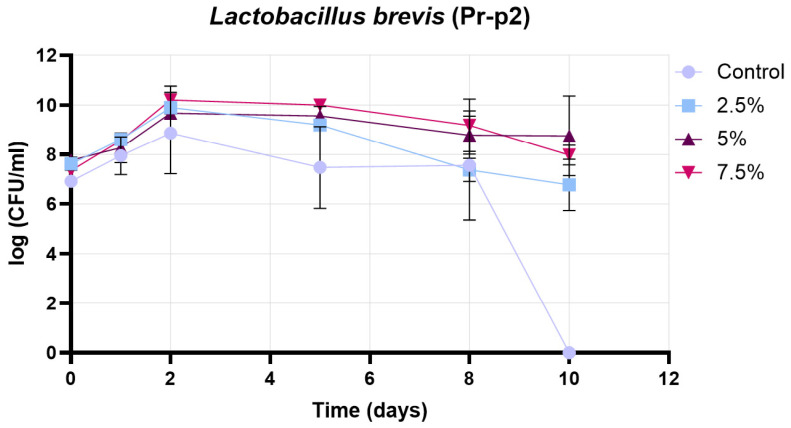
Results of growth curve determination for Pr-p2 strain (*Lactobacillus brevis*). Data are presented as mean ± SD; n = 3.

Figure 13 presents the growth curve of the Pr-p2 strain (*Lactobacillus brevis*) under four different conditions: control, 2.5% treatment, 5% treatment, and 7.5% treatment. The initial cell counts were comparable across all groups (approximately 7.0–7.8 log CFU/mL). During the early growth phase (days 0–2), all treatments showed an increase in viable cell numbers, with the highest peaks observed in the 7.5% (~10.2 log CFU/mL) and 5% (~9.8 log CFU/mL) treatments on day 2. The 2.5% treatment reached a slightly lower peak (~9.5 log CFU/mL), while the control group exhibited the lowest maximum (~8.9 log CFU/mL). From days 2 to 5, a gradual decline in cell counts was observed in all groups, with the control and 2.5% treatments showing a more pronounced decrease compared to the 5% and 7.5% treatments. Between days 5 and 8, the control group maintained only moderate viability (~7.9–8.0 log CFU/mL), while the 2.5% treatment continued to decline to ~7.6 log CFU/mL. In contrast, the 5% and 7.5% treatments retained higher viable counts (~8.9–9.3 log CFU/mL) during this period, suggesting a better protective effect. In the decline phase (days 8–10), the control group’s cell count dropped sharply to zero, whereas the 2.5% treatment ended at ~6.6 log CFU/mL. The 5% and 7.5% treatments demonstrated the highest survival, with final values of ~8.8 log CFU/mL and ~8.0 log CFU/mL, respectively. Overall, the results indicate that supplementation at 5% and 7.5% concentrations significantly enhanced both the peak growth and the long-term viability of the Pr-P2 strain, whereas the control and 2.5% treatments were less effective in maintaining cell counts throughout the experimental period.

## 4. Discussion

The present study comprehensively evaluated the physicochemical properties, encapsulation efficiency, release profile, biological safety, and functional effects of *Salvia officinalis* extract encapsulated in alginate-based microparticles, with the addition of inulin and pectin as prebiotic components.

The pursuit of effective delivery systems for bioactive phytochemicals remains a cornerstone of modern pharmaceutical and nutraceutical research, with the goal of enhancing their bioavailability, stability, and targeted action. In this study, alginate-based microbeads were meticulously formulated and characterized to co-encapsulate *Salvia officinalis* extract alongside the prebiotics inulin and pectin.

The initial characterization via Scanning Electron Microscopy (SEM) provided crucial insights into the physical attributes of the lyophilized *Salvia*-loaded alginate microparticles (Figure 1). The irregular, angular morphologies with faceted, wrinkled, and folded surfaces observed are typical of alginate beads produced by ionic gelation and subsequently freeze-dried. This surface architecture likely results from the collapse of the polymeric network during rapid water removal in lyophilization, producing a compact, non-spherical form. The occasional presence of calcium chloride crystals, arising from residual crosslinking agent, is consistent with previous reports and may slightly modify the surface chemistry [52,53]. Such morphological features are not merely esthetic descriptors; they have practical implications for the beads behavior in biological systems. Surface roughness and texture can directly affect mucoadhesion, cellular interaction, and drug release kinetics. For example, a rougher surface increases the available contact area and may promote stronger adhesion to mucosal tissues or enhance uptake by epithelial cells [54,55]. Similarly, microfolds and irregularities may create diffusion pathways that accelerate drug release, while smoother surfaces tend to support a more sustained release profile. These relationships have been reported in other microencapsulation studies and suggest that the observed wrinkled, irregular surfaces could facilitate a faster initial release phase followed by controlled diffusion from the interior matrix [56,57,58]. The incorporation of inulin and pectin at relatively high concentrations can indeed influence bead formation, alginate crosslinking, and the resulting structural properties. Pectin, being present at twice the concentration of alginate, can interact with calcium ions during gelation, potentially modifying the crosslinking density and creating a more heterogeneous gel network. Inulin, as a soluble polysaccharide, can increase the viscosity of the pre-gel solution, which may affect droplet formation during extrusion, bead size, and sphericity. Despite these potential effects, our results indicate that the combination of alginate with pectin and inulin produced stable beads with satisfactory encapsulation efficiency and controlled release profiles. The prebiotics may also contribute to gel reinforcement through polymer–polymer interactions, improving mechanical stability while preserving the protective effect of the alginate matrix. These considerations align with previous studies showing that high pectin content can slightly reduce bead compactness but overall enhances gel elasticity, whereas inulin increases solution viscosity and promotes more uniform bead formation [59].

The particle size range of approximately 235–314 µm, with minimal difference between empty (273 ± 12.5 µm) and S. officinalis- and prebiotics-loaded beads (275 ± 14.03 µm), falls within a size window considered favorable for oral administration and potential colonic targeting. The lack of statistically significant size change upon loading indicates that the incorporation of sage extract, inulin, and pectin does not disrupt the bead formation process.

The encapsulation efficiency (EE) is a critical metric for any drug delivery system, reflecting its ability to effectively entrap the active pharmaceutical ingredient. Our microbeads demonstrated an exceptional EE, consistently exceeding 90% (specifically 94 ± 0.57%) for the *S. officinalis* extract. This remarkably high retention rate, verified spectrophotometrically, attests to the optimized formulation and encapsulation process.

Several factors likely contribute to this high EE. Firstly, the ionic gelation of alginate with calcium ions creates a robust three-dimensional network that physically entraps the sage extract and prebiotic components. Liang et al. described that the presence of polysaccharides such as inulin and pectin enhances matrix density and reduces leakage, consistent with improved encapsulation performance in polysaccharide matrices [59]. The high EE is paramount, as it ensures maximal delivery of bioactive compounds, potentially reducing the required dose and minimizing waste [60,61]. Similarly, Park et al. emphasized that high EE values are essential for maintaining therapeutic agent stability and consistent dosing in delivery systems. Compared to other encapsulation techniques or less optimized alginate systems, which often report EEs in the range of 50–80% for various phytochemicals, the present formulation’s EE is notably superior [62,63,64]. Li, M., et al. (2016) [65]: In their study, alginate beads blended with additional polysaccharides (including cellulose powder, nanocellulose, starch, or xylan) and kaolin were produced by Ca^2+^ crosslinking. The addition of these polysaccharides—often at concentrations two to five times higher than alginate—enhanced the mechanical strength and modified release properties, indicating improved matrix density and controlled leakage [65].

Abka-Khajouei, R., et al. (2022): This comprehensive review details the reinforcement of Ca^2+^-crosslinked alginate hydrogels with larger relative amounts of various polysaccharides and their impact on improved mechanical and barrier properties of the beads for biomedical and pharmaceutical uses [66].

Additional summaries can be found in recent reviews of polysaccharide-based encapsulation systems, where blending alginate with higher ratios of polymers such as pectin or inulin is reported to increase bead density and minimize substance leakage due to synergistic network formation [67].

A study by Atia et al. (2016) [68] explored the effect of adding inulin to alginate beads at concentrations up to 20% (*w*/*v*). The results indicated that higher inulin concentrations improved the mechanical properties and acid resistance of the beads, suggesting that inulin contributes to the structural integrity of the alginate matrix [68].

These works confirm that using high ratios of secondary polysaccharides with alginate and Ca^2+^ crosslinking is a well-documented strategy for tailoring structural and functional properties of beads, including matrix density and payload retention.

The swelling behavior of hydrogel-based delivery systems like alginate beads is a pivotal characteristic, profoundly influencing drug release mechanisms, mucoadhesion, and transit time in the gastrointestinal tract [61,69]. Our investigation into the equilibrium water uptake (EWU) after 24 h (Figure 2) revealed that beads containing *Salvia officinalis*, inulin, and pectin absorbed significantly more water—around 90%—than empty beads or beads containing single prebiotic components. This suggests increased hydrophilicity and porosity due to the incorporation of these hydrophilic polysaccharides, which is in agreement with established reports indicating that prebiotic polysaccharides improve swelling capacity and thus the release kinetics of encapsulated compounds [70]. The phenolic compounds present in the *S. officinalis* extract, with multiple hydroxyl groups, likely further enhance water absorption through hydrogen bonding. A swollen hydrogel matrix with larger mesh size facilitates the diffusion of encapsulated compounds and may prolong gastric residence time or enhance interaction with the mucosal layer, potentially improving absorption. The enhanced swelling is therefore expected to facilitate controlled and sustained release in gastrointestinal conditions [71,72,73]. The enhanced swelling behavior observed in the sage-loaded beads is likely the result of a synergistic effect between the prebiotic polysaccharides and the *S. officinalis* extract. The hydrophilic polysaccharides, inulin and pectin, increase the water absorption capacity and porosity of the bead matrix, as reported in previous studies indicating that prebiotic polysaccharides improve swelling and modulate release kinetics [68]. In addition, the phenolic compounds present in *S. officinalis*, which contain multiple hydroxyl groups, can further enhance water uptake through hydrogen bonding. The combination of these effects results in a swollen hydrogel matrix with larger mesh size, facilitating the diffusion of encapsulated compounds, potentially prolonging gastric residence time, enhancing interaction with the mucosal layer, and promoting controlled and sustained release under gastrointestinal conditions [74,75].

The in vitro release study provided a comparative view of carnosol release from alginate beads containing *S. officinalis* and prebiotics. Alginate is known to be relatively stable at low pH, shrinking rather than dissolving, which limits the premature release of encapsulated compounds. This protection is vital for acid-labile drugs or for compounds intended for release further down the GI tract [61,76]. At neutral to alkaline pH values, typical of the intestinal environment, alginate microbeads undergo substantial swelling due to ion exchange processes. Specifically, calcium ions, which crosslink the alginate chains forming the gel matrix, are exchanged with monovalent ions such as sodium present in the intestinal fluid. This exchange weakens the “egg-box” structure of the alginate gel, causing its gradual erosion and partial dissolution. Furthermore, ions capable of chelating calcium, including phosphate and citrate ions commonly found in simulated intestinal fluids, accelerate this process and lead to gel breakdown. As a result, the microbeads progressively swell and erode, facilitating the sustained release of the encapsulated compounds [77,78]. Although the reviewer correctly noted that the formulation process involves dispersing the extract in aqueous solution, it should be emphasized that the sage extract is only slightly water-soluble, rather than highly soluble. During bead preparation, the extract is not present as a fully dissolved solute but rather as a suspension, with part of the phenolic fraction entrapped within the polymer matrix. Consequently, when placed in aqueous medium, the extract does not immediately leach out from the HPMC capsules. Instead, its limited solubility, together with its interaction with the alginate–prebiotic bead network, slows down dissolution and contributes to the observed controlled release behavior.

The seemingly contradictory descriptions actually reflect the dual role of prebiotic polysaccharides such as inulin and pectin in the bead matrix. On one hand, their hydrophilic nature increases water uptake and porosity, which loosens the surface structure and facilitates swelling, thereby promoting the release of encapsulated compounds. On the other hand, once hydrated, these polysaccharides form gel-like networks that reinforce the bead structure, preventing premature disintegration and allowing a more controlled and sustained release. In other words, prebiotics initially enhance swelling and diffusion, but they also contribute to maintaining matrix integrity during gastrointestinal transit. This dual functionality reconciles the two observations, as both effects occur simultaneously and are complementary rather than contradictory [79,80,81].

Consistent with these mechanisms, the release profile of carnosol from the alginate microbeads in simulated intestinal fluid (SIF) exhibited a marked sustainment, with approximately 85% cumulative release over 2 h. This contrasts with the free *S. officinalis* extract, which showed a more rapid and incomplete release. The sustained release is attributed to the protective alginate matrix, which controls diffusion and delays the release kinetics, thus offering a more controlled drug delivery system. This pH-responsive behavior is particularly beneficial for targeting delivery in the intestinal region, protecting the active compound from premature release or degradation in acidic gastric conditions [77,82].

These observations align with previous studies demonstrating that alginate beads behave as pH-sensitive reservoirs, swelling at higher pH and releasing their payload in a controlled manner, which can enhance the bioavailability and therapeutic efficacy of active agents [83]. In comparison, microbeads without prebiotics showed reduced drug release, mainly due to the absence of inulin and pectin prebiotics. The porosity and hydrophilicity of the matrix were increased by these prebiotics, thereby promoting water penetration and the diffusion of active ingredients. In their absence, a more compact microbead structure was formed, which limited carnosol release. This confirms a controlled-release mechanism, which is desirable for maintaining therapeutic levels over extended periods [84].

Furthermore, the incorporation of inulin and pectin into the bead matrix likely modulates release dynamics by reinforcing matrix integrity and influencing diffusion pathways. These prebiotic polysaccharides swell and form gel-like networks under physiological conditions, thus contributing to enhanced structural stability and controlled drug release [59,85,86].

The enzymatic stability assay assessed the resilience of *S. officinalis* and prebiotics-loaded alginate beads against pepsin in SGF (pH 1.2, Figure 4a) and pancreatin in SIF (pH 6.8, Figure 4b), compared to free sage extract. In the acidic, pepsin-containing simulated gastric fluid the encapsulated extract demonstrated significantly enhanced protection with notably less degradation. This suggests that, while carnosol and other extract components possess some inherent stability, the alginate matrix offers a substantial additional barrier that preserves the bioactive compounds more effectively under these conditions. The slightly superior stability of the beads could be attributed to the physical barrier of the alginate preventing enzyme access to the encapsulated material. The free extract underwent rapid and extensive degradation in SIF. This is a critical observation, as it implies that unprotected sage extract might lose a significant portion of its bioactivity before it can be absorbed or exert its effects in the lower GI tract. In contrast, the *S. officinalis*-loaded alginate beads exhibited significantly enhanced stability, with a much slower and more gradual degradation profile [86,87]. These findings align with prior studies where encapsulation enhanced the intestinal stability of phenolic compounds [88]. Carnosol, a phenolic diterpene, is known to be chemically unstable and prone to degradation under physiological conditions. In the presence of pancreatin, which contains esterases, lipases, and proteases, its degradation is likely accelerated through oxidative and hydrolytic processes. Pancreatic enzymes, particularly esterases and lipoxygenases, can catalyze reactions that destabilize diterpenoid phenolics, leading to cleavage of ester bonds, oxidation of hydroxyl groups, and subsequent structural breakdown [89]. Previous studies have also reported that carnosol and related polyphenols are unstable in intestinal environments due to enzymatic and oxidative stress, resulting in reduced bioactive concentrations after exposure to pancreatin-rich media [90,91].

The rapid loss of free carnosol in simulated intestinal fluid containing pancreatin can be attributed to enzyme-mediated degradation combined with intrinsic oxidative instability, highlighting the protective role of encapsulation. The stability assessment in this study focused on carnosol at 285 nm as a representative marker compound, given its abundance and well-established UV absorbance profile. While this provides a reliable indicator of the release kinetics, it does not comprehensively account for all phenolic constituents of the *S. officinalis* extract. However, the sustained release profile observed from the inulin–pectin–alginate beads suggests that the encapsulation matrix provided protection against degradation under gastrointestinal conditions, since premature leakage or loss of carnosol was not detected.

Carnosol itself is known to be relatively stable under neutral pH and oxidative environments when appropriately encapsulated [92]. Reports on polyphenol-loaded alginate and pectin hydrogels confirm that these matrices can prevent the degradation of multiple phenolic compounds beyond the single marker measured, by limiting oxygen exposure and controlling pH-driven hydrolysis [93,94,95]. Thus, while only carnosol was quantified spectrophotometrically, the controlled-release behavior, absence of burst degradation, and literature evidence of prebiotic polysaccharide matrices stabilizing phenolic compounds together support the conclusion that carnosol and other extract constituents were inherently stable under the tested conditions.

The MTT viability assay assessed the cytocompatibility of both unformulated sage extract and the formulated microbeads on Caco-2 cells, a standard model for intestinal epithelium. The free extract, at all tested concentrations, maintained cell viability between 70% and 95%, indicating relatively low intrinsic cytotoxicity. This is a positive finding, suggesting that the extract is reasonably well tolerated by intestinal cells. Similarly, the formulated beads demonstrated low cytotoxicity across various concentrations, with cell viability consistently remaining high. Importantly, for most concentrations, viability exceeded the 70% threshold commonly considered indicative of acceptable cytocompatibility in in vitro screening [96].

The treatment of Caco-2 monolayers resulted in a progressive increase in TEER values over the measurement period, indicating a tightening of the paracellular barrier. This enhancement in barrier function can be attributed to the synergistic effects of the formulation components. As it is described in the literature, the polysaccharides (alginate, inulin, and pectin) support epithelial integrity by forming a protective coating on the apical cell surface and stabilizing tight junction proteins such as occludin, claudins, and ZO-1 [97,98,99]. At the same time, the polyphenolic constituents of *S. officinalis*, including rosmarinic acid and carnosol, possess strong antioxidant and anti-inflammatory properties, which may counteract oxidative or inflammatory stress-induced disruption of the epithelial barrier [7,10,11].

Carnosol exhibits a highly lipophilic character and low aqueous solubility, making paracellular transport unlikely [100]. Carnosol, with a molecular weight of approximately 330 g/mol, can readily pass through cell membranes via passive transcellular diffusion [101]. These properties are conducive to passive transcellular diffusion, whereby it traverses the lipid bilayer along its concentration gradient without the necessity for energy input or transporter proteins [97,102]. This transport route is minimally influenced by changes in TEER, as it does not rely on paracellular tight junction openings. Several in vitro and in silico studies classify carnosol as a highly lipophilic antioxidant predominantly absorbed via passive transcellular diffusion, with possible, but limited, interaction with efflux systems such as P-glycoprotein [103,104]. The combination of the physical barrier reinforcement by polysaccharides and the bioactive, lipophilic antioxidant properties of carnosol and other polyphenols likely accounts for the observed sustained increase in TEER, suggesting that the formulation can both strengthen epithelial integrity and allow effective absorption of certain lipophilic constituents [105,106].

The in vitro permeability assay, designed to measure the transport of *S. officinalis* bioactives, revealed that our formulation not only maintained but increased TEER values, indicating tighter paracellular junctions. Under normal circumstances, a rise in TEER would be expected to reduce the movement of compounds that cross via the paracellular pathway. However, a significant increase in the permeation of the bioactive components of the sage into the basolateral compartment was still observed at 2, 4, and 24 h compared to control beads. This is explained by the transport characteristics of carnosol and similar lipophilic constituents, which primarily cross the epithelium via the passive transcellular route, moving through the lipid bilayer rather than between cells [103,104]. Enhanced permeability could be attributed to the sustained release of bioactives and the possible interaction of prebiotics with tight junctions, which may increase paracellular transport [107]. Since this pathway does not require fully open tight junctions, higher TEER values do not hinder compound uptake. On the contrary, the formulation may enhance absorption by protecting these compounds from degradation in the apical compartment and maintaining a high concentration gradient at the cell membrane. Furthermore, its controlled release profile supports steady transcellular transport over time.

The antioxidant potential of the formulations was quantitatively assessed using the DPPH radical scavenging assay. The results were compelling: the *S. officinalis*- and prebiotics-containing formulation exhibited a significant 65% DPPH scavenging capacity. This level of activity is substantial and positions the formulation as a potent source of natural antioxidants. *S. officinalis* is well-known for its rich content of phenolic compounds, such as carnosic acid, carnosol, and rosmarinic acid, which are potent antioxidants due to their ability to donate hydrogen atoms or electrons to stabilize free radicals [7,8,9]. The encapsulation process appears to have successfully preserved this inherent antioxidant activity. Comparing this to Trolox, a water-soluble vitamin E analog used as a positive control, the 65% activity of the formulation is noteworthy. While not reaching the level of pure Trolox, it surpasses the antioxidant capacity reported for many crude plant extracts or even some isolated natural compounds [108]. The presence of prebiotics like inulin and pectin is unlikely to directly contribute significantly to DPPH radical scavenging, as their primary roles are structural and as substrates for gut microbiota. Therefore, the observed antioxidant activity can be primarily attributed to the bioactive compounds encapsulated from *S. officinalis* extract. Although the DPPH assay shows lower apparent antioxidant activity for the microbead samples compared to the free extract, this is largely due to the lower fraction of actual extract in the bead mass and the removal of polymer particles during filtration. Importantly, the formulation itself is highly effective, the encapsulation successfully preserves carnosol and other phenolics, enabling sustained release and maintaining high antioxidant potential over time. Therefore, the observed lower immediate activity does not reflect a lack of efficacy but rather the controlled-release behavior and successful stabilization provided by the alginate–prebiotic matrix [109].

Similarly, the CUPRAC assay revealed significant ROS inhibition, with microbeads achieving around 35–40% and the free extract approximately 65–70%. These results highlight the strong intrinsic antioxidant properties of *S. officinalis*, attributable to its rich profile of polyphenolic compounds, such as rosmarinic acid and carnosic acid, which are known to act as efficient electron and hydrogen donors [7,8,9]. The comparatively lower activity of the microbead formulations likely reflects the protective encapsulation matrix, which, while conferring stability and controlled release, can transiently limit the immediate availability of active phytochemicals. Nevertheless, the observed activity in encapsulated forms confirms that the microbeads retain substantial antioxidant functionality, supporting their potential use in delivery systems where stability and sustained release are advantageous. Taken together, the dual-assay data demonstrate that *S. officinalis* represents a potent natural antioxidant source in both free and encapsulated states, with the choice of formulation depending on whether maximal radical scavenging or prolonged functional stability is desired.

Chronic inflammation is a key driver of many gastrointestinal diseases. As described by Abd El-Hack et al. [22], *S. officinalis* and its bioactive compounds exhibit diverse health benefits, including epigenetic modulation and anti-inflammatory effects, which contribute to both human and livestock nutrition. The anti-inflammatory potential of *S. officinalis* is well documented, particularly its ability to inhibit pro-inflammatory pathways such as NF-κB signaling and reduce cytokine production [22]. In our study, the anti-inflammatory efficacy of the formulated microbeads was assessed by measuring the production of the pro-inflammatory cytokines TNF-α and IL-1β in Caco-2 cells stimulated with hydrogen peroxide (H_2_O_2_), a known inducer of oxidative stress and inflammation. The results demonstrated a significant decrease in both TNF-α and IL-1β levels following treatment with the formulation. Two experimental protocols were used: a therapeutic approach, where inflammation was induced prior to treatment, and a simultaneous approach, where inflammation and treatment proceeded concurrently. The therapeutic potential was particularly evident, with a marked reduction in cytokine secretion when the formulation was applied after the H_2_O_2_-induced inflammatory stimulus. This highlights the ability of the formulation to effectively dampen established inflammatory responses, consistent with the mechanisms described by Ghasemian et al. [110].

Interestingly, the combined treatment protocol also yielded a moderate but statistically significant reduction in inflammatory markers, nearly matching the post-treatment effect, suggesting the formulation may also provide preventive benefits against inflammatory triggers. The encapsulation of *S. officinalis* extract within the alginate–prebiotic matrix likely enhances their stability and cellular delivery, supporting their anti-inflammatory activity at the intestinal level. These findings corroborate the traditional use of sage as an anti-inflammatory agent and provide mechanistic insight into its efficacy in mitigating gut inflammation [110].

The collective evidence positions these microbeads as a multifaceted delivery platform. As described by Roberfroid et al., the incorporation of prebiotics such as inulin and pectin offers significant potential for synergistic benefits by promoting a healthy gut microbiome, which can positively influence both local and systemic health [111]. Although this aspect was not directly explored in the current cellular studies, Gibson and Roberfroid also emphasized that dietary modulation of the colonic microbiota through prebiotics plays a crucial role in maintaining gut homeostasis and overall well-being [28,70]. Thus, the strategic inclusion of these prebiotic components in the microbeads may provide additional health advantages beyond the encapsulated bioactive delivery.

Our results showed that the effect of *S. officinalis*- and prebiotics-containing microbeads on Lactobacillus growth and long-term survival was both strain- and concentration-dependent. For *L. rhamnosus* (Br-p1) (Figure 11), 2.5% proved optimal for long-term viability, with a delayed growth peak but improved survival in later stages (days 6–10), likely due to bacterial adaptation to sage bioactives, as reported by Succi et al. (2017) and Su et al. (2007) [112,113]. For *L. plantarum* (45210) (Figure 12), 2.5% was also most effective; while 5% initially offered protection, cell counts dropped to zero by day 10, suggesting that higher doses may cause inhibitory effects over time [114]. In contrast, *L. brevis* (Pr-p2) (Figure 13) excelled at higher concentrations (5–7.5%), showing both the highest peak growth and exceptional long-term survival. This superior performance suggests an efficient utilization of sage-derived nutrients and a remarkable tolerance to potential inhibitory metabolites, in line with its documented resilience to stress [115,116]. These differences likely reflect strain-specific metabolic adaptations, differential utilization of sage-derived carbohydrates, and varying tolerance to antioxidant and antimicrobial components. The term “sage-derived carbohydrates” refers to the small amounts of naturally occurring sugars and polysaccharides present in the *S. officinalis* extract. These compounds can serve as additional carbon sources for bacterial growth, potentially influencing metabolism in a strain-specific manner. They are not the primary active components but may contribute marginally to energy utilization alongside other extract constituents such as phenolics [117,118].

Overall, the sage-based prebiotic supports bacterial survival beyond the stationary phase; however, the optimal concentration must be tailored for each strain to maximize functional benefits.

Our work underscores the value of developing multifunctional microencapsulation systems to improve the stability, bioavailability, and sustained release of bioactive compounds with limited inherent stability, such as *S. officinalis* extract. As reported in the literature, the synergistic inclusion of prebiotic components like inulin and pectin not only enhances the delivery matrix but also supports gut microbiota health, adding an additional therapeutic dimension [119]. It can be concluded that the encapsulation of *S. officinalis* extract within alginate–prebiotic microparticles provides a promising platform for overcoming challenges related to compound degradation and absorption.

## 5. Conclusions

In conclusion, this study demonstrates that alginate-based microparticles co-encapsulating *S. officinalis* extract with prebiotic polysaccharides inulin and pectin represent a promising multifunctional delivery platform. The microbeads exhibited high encapsulation efficiency, controlled and sustained release of carnosol, and enhanced stability against gastrointestinal degradation. The inclusion of prebiotics supported probiotic bacterial survival and contributed to the maintenance of intestinal barrier integrity, as evidenced by increased TEER values and reduced pro-inflammatory cytokine production in vitro. Moreover, the formulation displayed significant antioxidant and anti-inflammatory activities, aligning with traditional therapeutic uses of sage. Looking ahead, future research will focus on large-scale production and process optimization, including ensuring batch-to-batch consistency. Further in vivo and clinical studies are planned to validate the translational potential of this delivery system for managing gastrointestinal health and related inflammatory conditions. These efforts aim to bridge laboratory findings with practical therapeutic applications, expanding the use of alginate-based microparticles in targeted and controlled drug delivery. This study thus lays a solid foundation for continued development toward commercial and clinical translation.

## Data Availability

The data that support the findings of this study are available from the corresponding author (jozsa.liza@euipar.unideb.hu) with the permission of the head of the department, upon reasonable request.

## Supplementary Materials

The following supporting information can be downloaded at: https://www.mdpi.com/article/10.3390/pharmaceutics17101308/s1, Figure S1: Analysis and specification of the sage extract used in the study; Figure S2: SEM images (microcapsules with sage extract and prebiotics and empty microcapsules).
