# Supplementary material for: Formulation and Testing of Alginate Microbeads Containing Salvia officinalis Extract and Prebiotics
Source: Pharmaceutics. 2025 Oct 8;17(10):1308. doi: 10.3390/pharmaceutics17101308 (PMC12566799; doi:10.3390/pharmaceutics17101308)
Supplement: Supplementary file 1 [file pharmaceutics-17-01308-s001.zip › pharmaceutics-3869454-supplementary.pdf]

Supplementary material for:

Formulation and testing of alginate microbeads containing *Salvia officinalis* extract and prebiotics

Krisztina Bodnár [1,2], Pálma Fehér [1,2], Zoltán Ujhelyi [2,3], Ádám Haimhoffer [1,2], Boglárka Papp [1,2], Dávid Sinka [1,2], Csongor Freytag [4], Eszter Fidrus [4], Krisztina Szarka [5], Gábor Kardos [6,7], Fruzsina Nacsa [8], Ildikó Bácskay [1,2] and Liza Józsa [1,2,*]

1 Department of Pharmaceutical Technology, Faculty of Pharmacy, University of Debrecen, 4032 Debrecen, Hungary; bodnar.krisztina@pharm.unideb.hu (K.B.); feher.palma@pharm.unideb.hu (P.F.); haimhoffer.adam@euipar.unideb.hu (Á.H.); papp.boglarka@pharm.unideb.hu (B.P.); sinka.david@pharm.unideb.hu (D.S.); bacskay.ildiko@pharm.unideb.hu (I.B.); jozsa.liza@euipar.unideb.hu (L.J.)

2 Doctoral School of Pharmaceutical Sciences, University of Debrecen, 4032 Debrecen, Hungary

3 Department of Industrial Pharmaceutical Technology, Faculty of Pharmacy, University of Debrecen, Rex Ferenc Utca 1, H-4002 Debrecen, Hungary; ujhelyi.zoltan@pharm.unideb.hu (Z.U.)

4 Department of Bioinformatics, One Health Institute, Faculty of Health Sciences, University of Debrecen, 4032 Debrecen, Hungary; freytag.csongor@etk.unideb.hu (C.F.); fidrus.eszter@etk.unideb.hu (E.F.)

5 Department of Infection Control and Hospital Epidemiology, One Health Institute, Faculty of Health Sciences, University of Debrecen, 4032 Debrecen, Hungary; szkrisz@med.unideb.hu (K.Sz.)

6 Department of Planetary Health, One Health Institute, Faculty of Health Sciences, University of Debrecen, 4032 Debrecen, Hungary; kg@med.unideb.hu (G.K.)

7 Institute of Metagenomics, University of Debrecen, 4032 Debrecen, Hungary

8 MEDITOP Pharmaceutical Ltd., 2097 Pilisborosjeno, Hungary; fruzsina.nacsa@meditop.hu (F.N.)

* Correspondence: jozsa.liza@euipar.unideb.hu

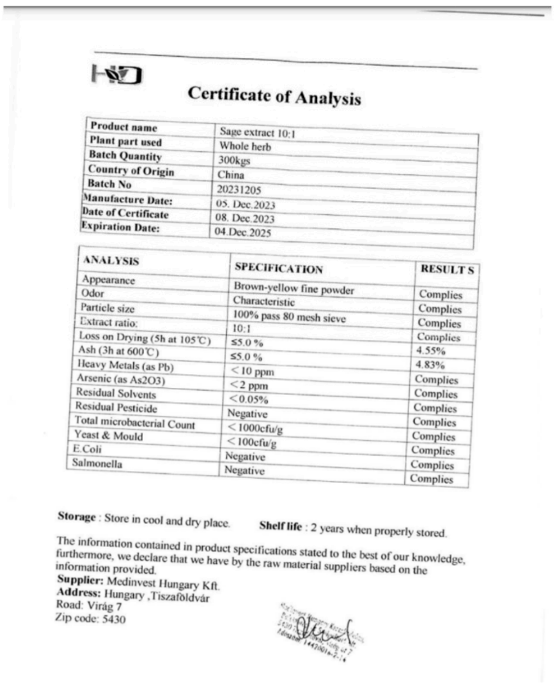

Certificate of Analysis

**Product name**	Sage extract 10:1
**Plant part used**	Whole herb
**Batch Quantity**	300kgs
**Country of Origin**	China
**Batch No**	20231205
**Manufacture Date:**	05. Dec.2023
**Date of Certificate**	08. Dec.2023
**Expiration Date:**	04.Dec.2025

ANALYSIS	SPECIFICATION	RESULT S
Appearance	Brown-yellow fine powder	Complies
Odor	Characteristic	Complies
Particle size	100% pass 80 mesh sieve	Complies
Extract ratio:	10:1	Complies
Loss on Drying (5h at 105℃)	≤5.0 %	4.55%
Ash (3h at 600℃)	≤5.0 %	4.83%
Heavy Metals (as Pb)	＜10 ppm	Complies
Arsenic (as As2O3)	＜2 ppm	Complies
Residual Solvents	＜0.05%	Complies
Residual Pesticide	Negative	Complies
Total microbacterial Count	＜1000cfu/g	Complies
Yeast & Mould	＜100cfu/g	Complies
E.Coli	Negative	Complies
Salmonella	Negative	Complies

Storage : Store in cool and dry place. **Shelf life** : 2 years when properly stored.

The information contained in product specifications stated to the best of our knowledge, furthermore, we declare that we have by the raw material suppliers based on the information provided.

Supplier: Medinvest Hungary Kft.
Address: Hungary ,Tiszaföldvár
Road: Virág 7
Zip code: 5430

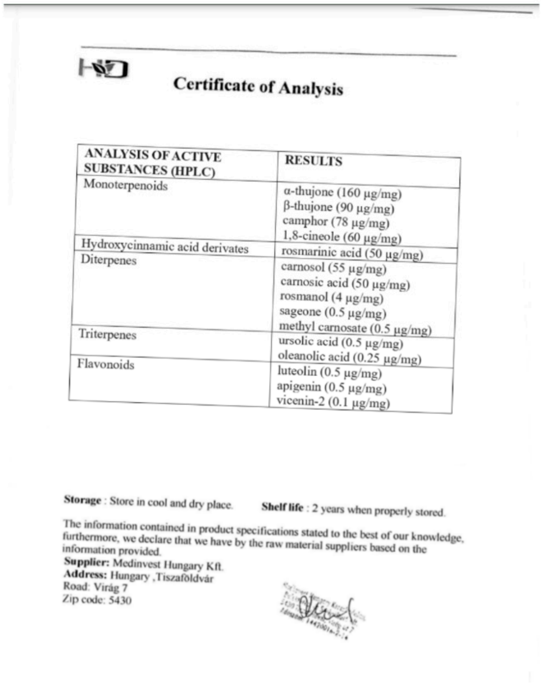

Certificate of Analysis

ANALYSIS OF ACTIVE SUBSTANCES (HPLC)	RESULTS
Monoterpenoids	α-thujone (160 μg/mg)<br>β-thujone (90 μg/mg)<br>camphor (78 μg/mg)<br>1,8-cineole (60 μg/mg)
Hydroxycinnamic acid derivates	rosmarinic acid (50 μg/mg)
Diterpenes	carnosol (55 μg/mg)<br>carnosic acid (50 μg/mg)<br>rosmanol (4 μg/mg)<br>sageone (0.5 μg/mg)<br>methyl carnosate (0.5 μg/mg)
Triterpenes	ursolic acid (0.5 μg/mg)<br>oleanolic acid (0.25 μg/mg)
Flavonoids	luteolin (0.5 μg/mg)<br>apigenin (0.5 μg/mg)<br>vicenin-2 (0.1 μg/mg)

Storage : Store in cool and dry place. **Shelf life** : 2 years when properly stored.

The information contained in product specifications stated to the best of our knowledge, furthermore, we declare that we have by the raw material suppliers based on the information provided.

Supplier: Medinvest Hungary Kft.
Address: Hungary ,Tiszaföldvár
Road: Virág 7
Zip code: 5430

Figure S1. Analysis and specification of the sage extract used in the study

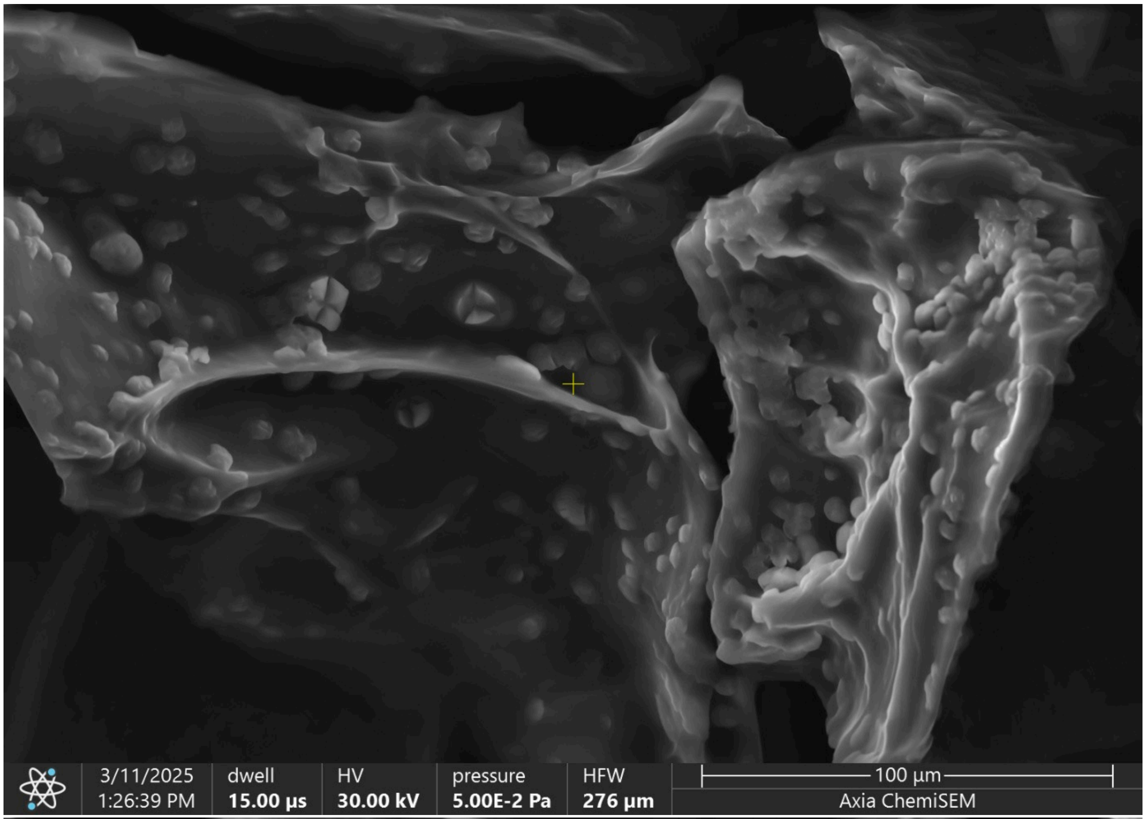
3/11/2025
1:26:39 PM
dwell
15.00 µs
HV
30.00 kV
pressure
5.00E-2 Pa
HFW
276 µm
100 µm
Axia ChemiSEM

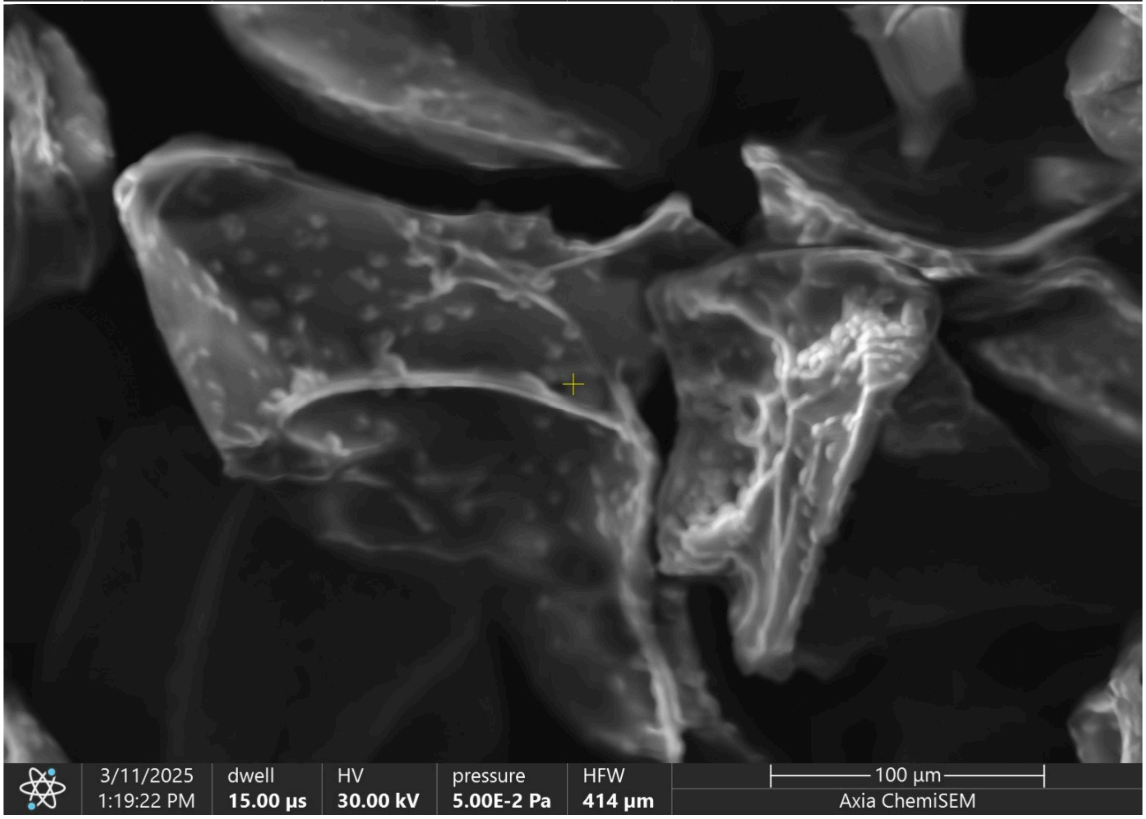
3/11/2025
1:19:22 PM
dwell
15.00 µs
HV
30.00 kV
pressure
5.00E-2 Pa
HFW
414 µm
100 µm
Axia ChemiSEM

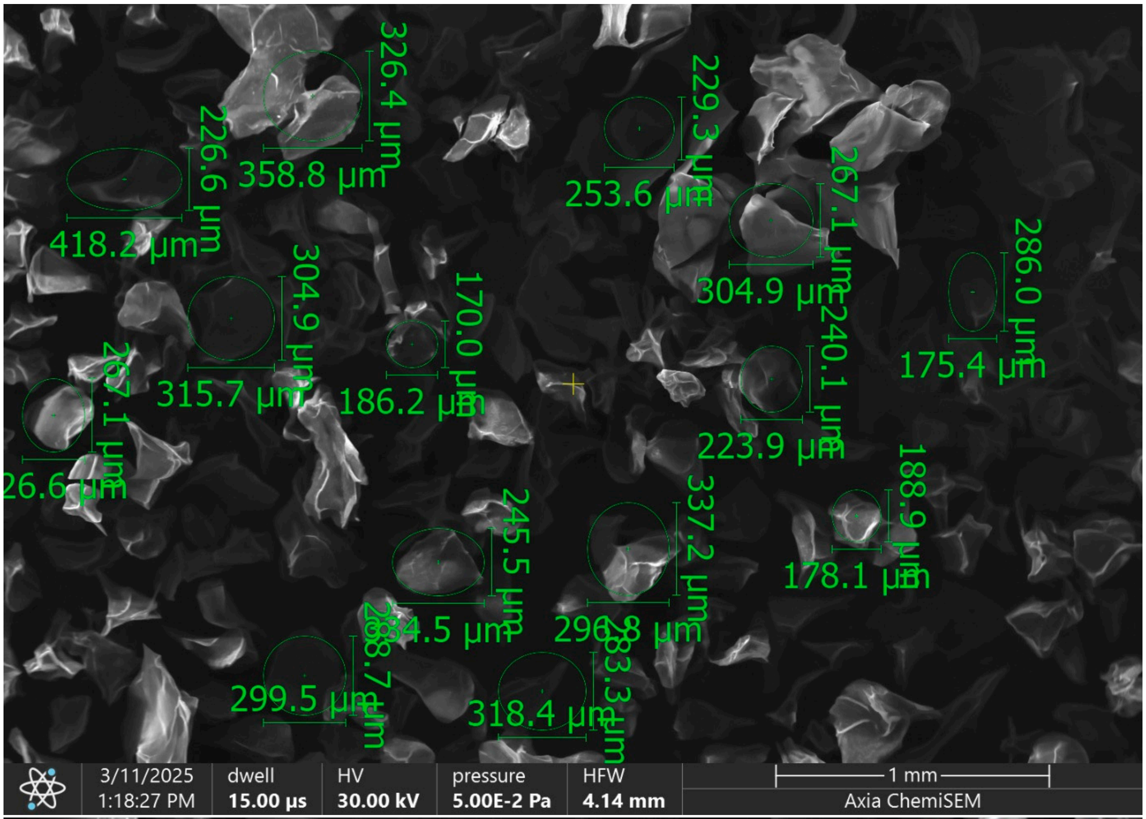

326.4 µm
358.8 µm
226.6 µm
418.2 µm
229.3 µm
253.6 µm
267.1 µm
304.9 µm
286.0 µm
175.4 µm
304.9 µm
315.7 µm
170.0 µm
186.2 µm
240.1 µm
223.9 µm
267.1 µm
188.9 µm
178.1 µm
245.5 µm
337.2 µm
299.5 µm
318.4 µm
3/11/2025
1:18:27 PM
dwell
15.00 µs
HV
30.00 kV
pressure
5.00E-2 Pa
HFW
4.14 mm
1 mm
Axia ChemiSEM

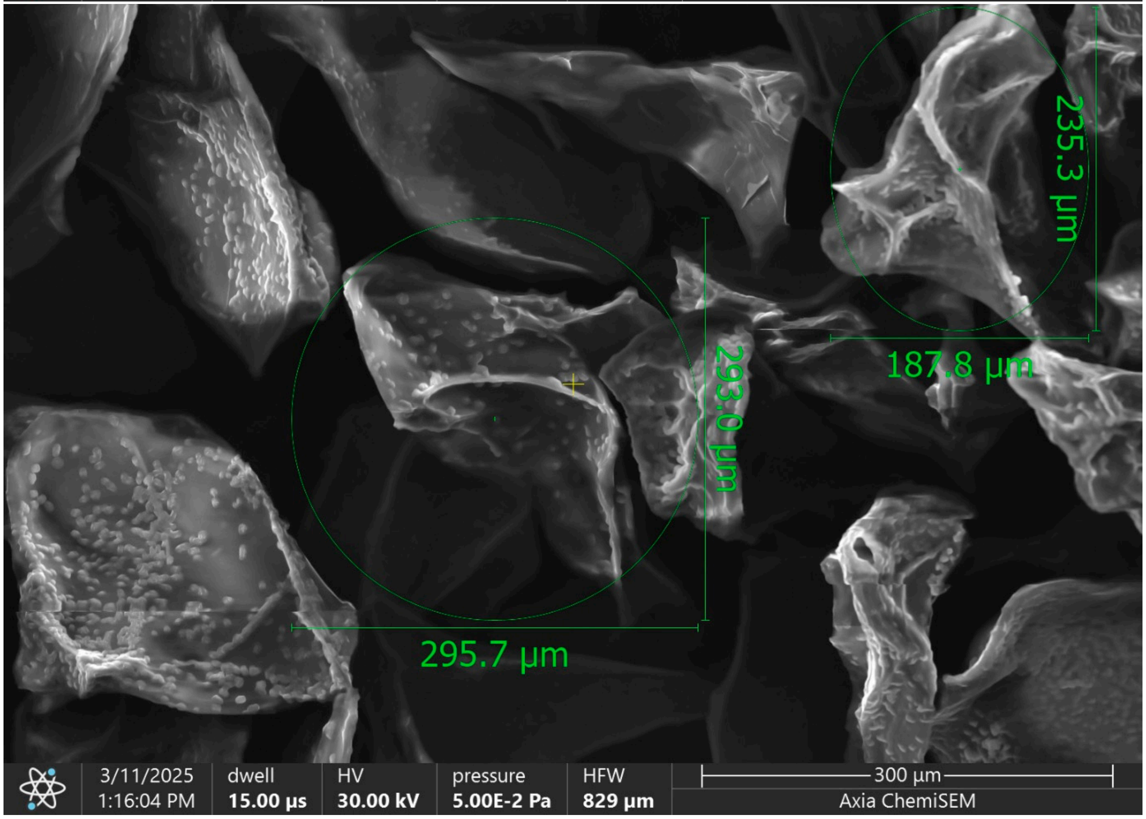

235.3 µm
187.8 µm
293.0 µm
295.7 µm
3/11/2025
1:16:04 PM
dwell
15.00 µs
HV
30.00 kV
pressure
5.00E-2 Pa
HFW
829 µm
300 µm
Axia ChemiSEM

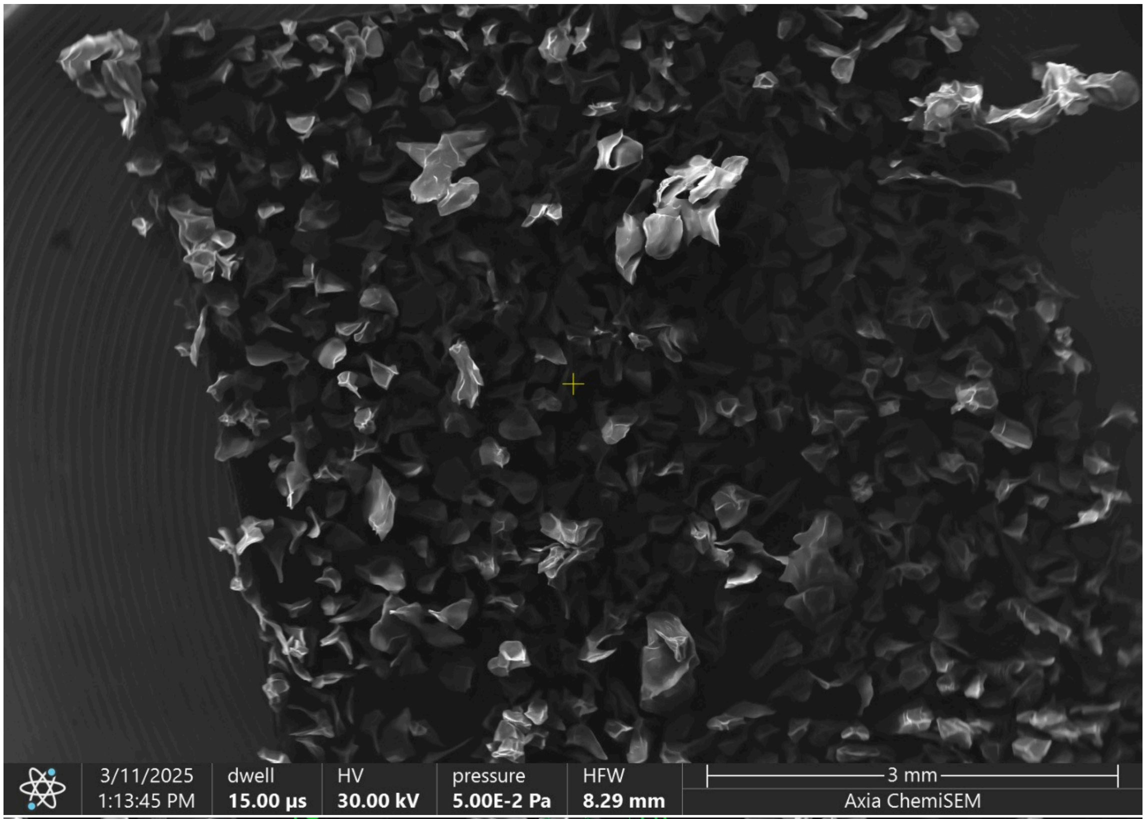
3/11/2025
1:13:45 PM
dwell
15.00 µs
HV
30.00 kV
pressure
5.00E-2 Pa
HFW
8.29 mm
3 mm
Axia ChemiSEM

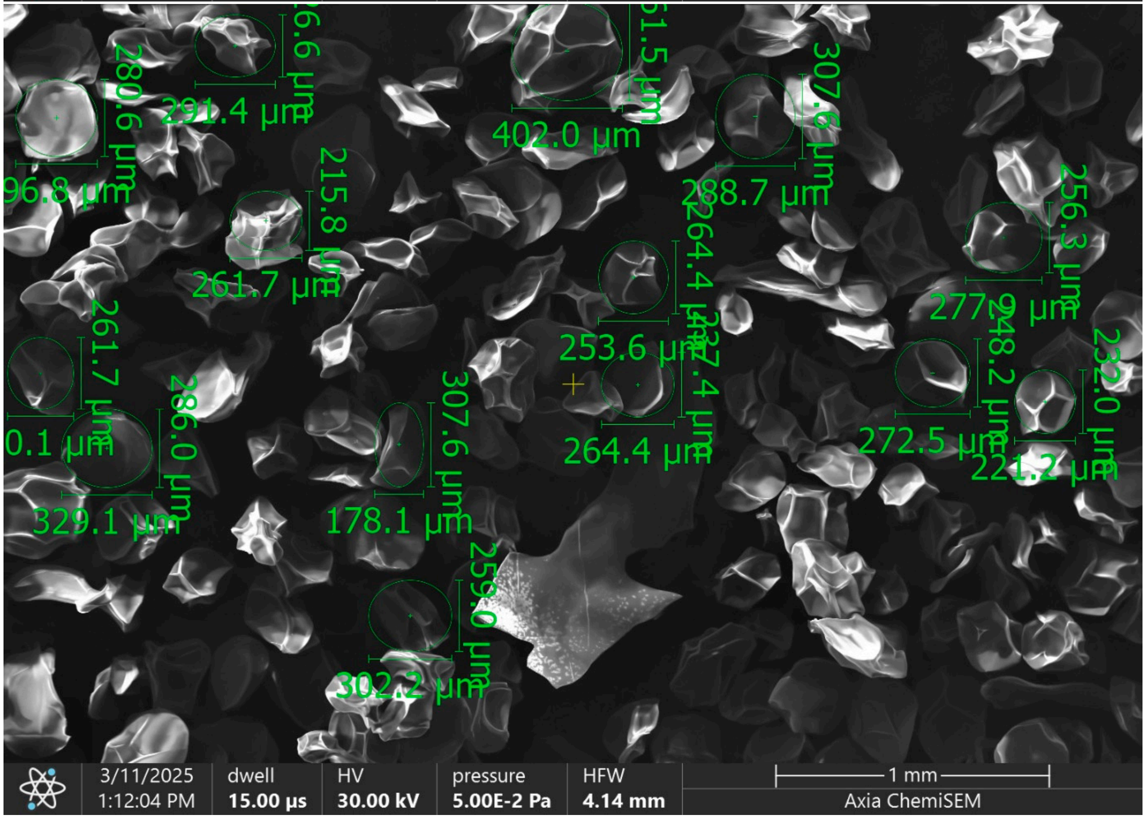
291.4 µm
402.0 µm
288.7 µm
215.8 µm
261.7 µm
256.3 µm
253.6 µm
261.7 µm
286.0 µm
307.6 µm
264.4 µm
272.5 µm
221.2 µm
232.0 µm
329.1 µm
178.1 µm
259.0 µm
302.2 µm
3/11/2025
1:12:04 PM
dwell
15.00 µs
HV
30.00 kV
pressure
5.00E-2 Pa
HFW
4.14 mm
1 mm
Axia ChemiSEM

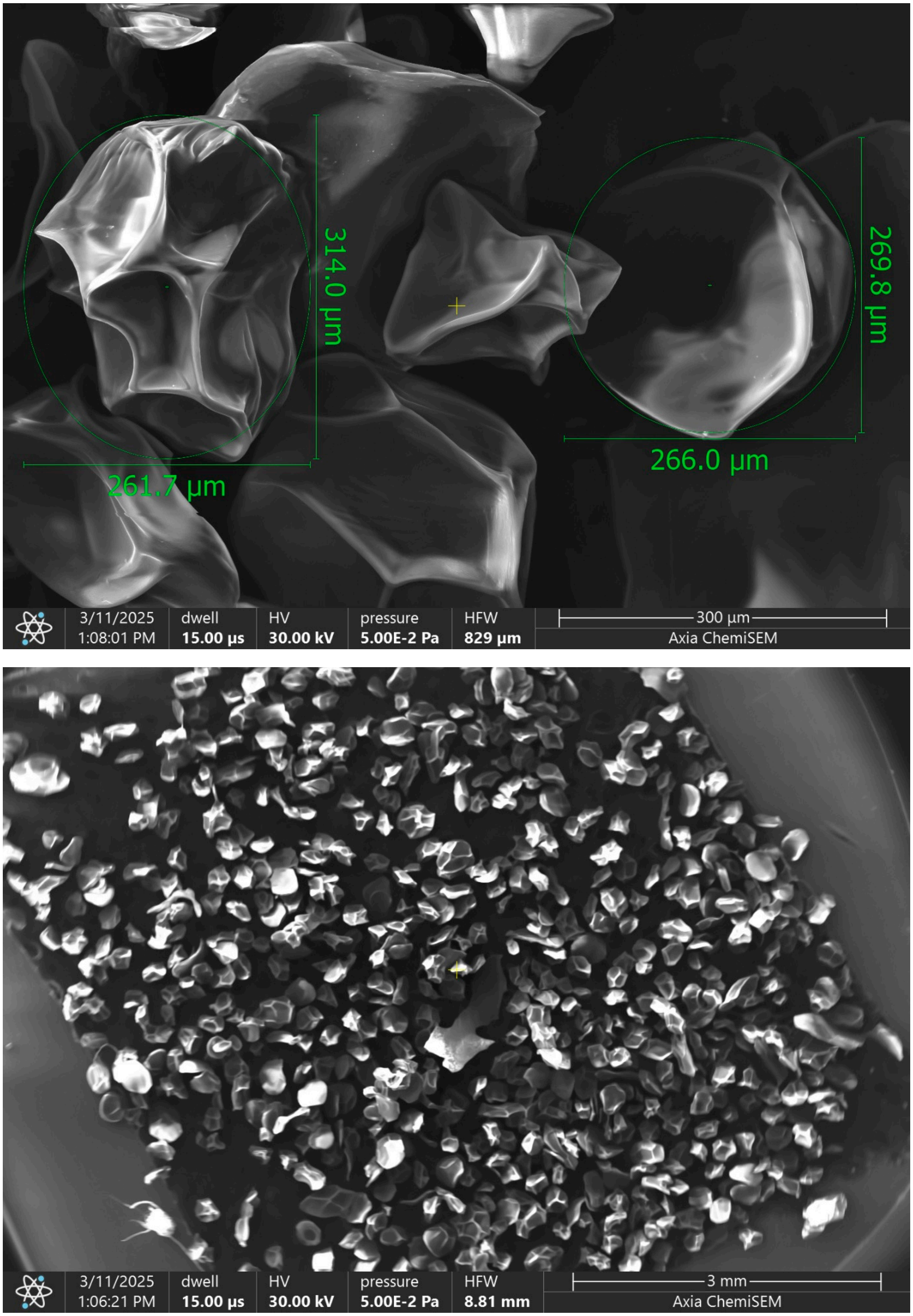

Figure S2. SEM images (microcapsules with sage extract and prebiotics and empty microcapsules)
